# A Triazolium‐Anchored Self‐Immolative Linker Enables Self‐Assembly‐Driven siRNA Binding and Esterase‐Induced Release

**DOI:** 10.1002/chem.202203311

**Published:** 2022-12-16

**Authors:** Selina Hollstein, Lamiaa M. A. Ali, Maëva Coste, Julian Vogel, Nadir Bettache, Sébastien Ulrich, Max von Delius

**Affiliations:** ^1^ Institute of Organic Chemistry Ulm University Albert-Einstein-Allee 11 89081 Ulm Germany; ^2^ Institut des Biomolécules Max Mousseron (IBMM) CNRS Université de Montpellier, ENSCM Montpellier France; ^3^ Department of Biochemistry Medical Research Institute University of Alexandria 21561 Alexandria Egypt

**Keywords:** amphiphiles, click chemistry, drug delivery, self-assembly, siRNA delivery

## Abstract

The increased importance of RNA‐based therapeutics comes with a need to develop next‐generation stimuli‐responsive systems capable of binding, transporting and releasing RNA oligomers. In this work, we describe triazolium‐based amphiphiles capable of siRNA binding and enzyme‐responsive release of the nucleic acid payload. In aqueous medium, the amphiphile self‐assembles into nanocarriers that can disintegrate upon the addition of esterase. Key to the molecular design is a self‐immolative linker that is anchored to the triazolium moiety and acts as a positively‐charged polar head group. We demonstrate that addition of esterase leads to a degradation cascade of the linker, leaving the neutral triazole compound unable to form complexes and therefore releasing the negatively‐charged siRNA. The reported molecular design and overall approach may have broad utility beyond this proof‐of‐principle study, because the underlying CuAAC “click” chemistry allows bringing together three groups very efficiently as well as cleaving off one of the three groups under the mild action of an esterase enzyme.

## Graphical abstract

**Self‐assembled** triazolium‐based amphiphiles lose the ability to bind siRNA upon addition of esterase, due to the degradation of the polar head group and conversion to a neutral triazole.

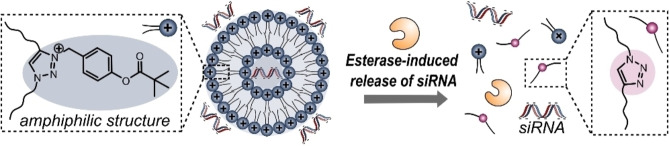

## Introduction

After a bumpy road paved with hopes and failures, patients can today benefit from small interfering RNA (siRNA) drugs[^1^, ^2^] and messenger RNA (mRNA) vaccines.[^3^, ^4^] In 2018, the first‐of‐its‐kind siRNA‐based drug Patisiran was FDA‐approved, followed by three other RNA interference‐based therapeutics in 2019, 2020 and 2021 (Givosiran, Lumasiran, Inclisiran).[^5^, ^6^] In 2020, the FDA granted an emergency use authorization of mRNA vaccines to fight the Covid‐19 pandemic,^[7]^ and further RNA‐based drugs are currently in clinical trials.[^8^, ^9^]

The success of these RNA‐based therapeutics relies largely on the development of lipid nanoparticle nanovectors such as micelleplexes[^10^, ^11^, ^12^, ^13^] and liposomes[^14^, ^15^, ^16^, ^17^] which protect RNAs from enzymatic degradation by nucleases, while also promoting (targeted) cell internalization.[^18^, ^19^, ^20^, ^21^, ^22^, ^23^] The composition of those FDA‐approved four‐component (lipid‐PEG, helper lipid, cholesterol, ionizable lipid) nanoparticles is crucial for effective cell delivery. In particular, the nature of the ionizable lipid has been optimized to greatly improve their delivery efficacy through an enhancement in endosomal escape – two‐armed cylindrical or inverted cone structures with packing parameter greater than 1/2 being a must.[^24^, ^25^, ^26^, ^27^]

However, these multi‐component formulations must be preserved at (ultra)low temperatures for storage and distribution to preserve their structure (current limit of 1–3 months at 2–8 °C for the mRNA Covid‐19 vaccines[^28^, ^29^]). Therefore, a current challenge in the field is to make these lipid nanoparticles more stable while keeping their dynamic nature which is key to deliver the nucleic acid payload. In other works, self‐assembly has to be dynamically expressed, turned on to stabilize nucleic acid complexes and turned off to trigger nucleic acid release.[^30^, ^31^, ^32^, ^33^, ^34^] In this line, the development of responsive systems is of current interest for the next generation of lipid nanoparticles.

Several reports aimed at addressing this challenge, in particular through the design of i) biodegradable lipid nanoparticles that degrade at the acidic pH found in late endosomes or in the intracellular reductive environment,[^35^, ^36^, ^37^, ^38^, ^39^] or ii) pH‐responsive switchable lipids.[^40^, ^41^, ^42^, ^43^] In addition to these endogenous pH and redox effectors, enzymes also represent a trigger of choice for controlled drug delivery.[^44^, ^45^, ^46^] Hitherto, a limited number of cases have reported on enzyme‐responsive RNA delivery using peptide conjugates,^[47]^ modified siRNA,^[48]^ and ester‐based amphiphilic dendrimers.^[49]^

Herein, we report the synthesis of amphiphiles with positively charged triazolium head groups that self‐assemble into vesicular structures and are able to complex siRNA. After addition of a stimulus (here: esterase) the polar head group of the amphiphiles degrades irreversibly by forming a neutral triazole and siRNA gets released.

## Results and Discussion

### Design of amphiphiles and aggregation behavior

**Figure 1:**
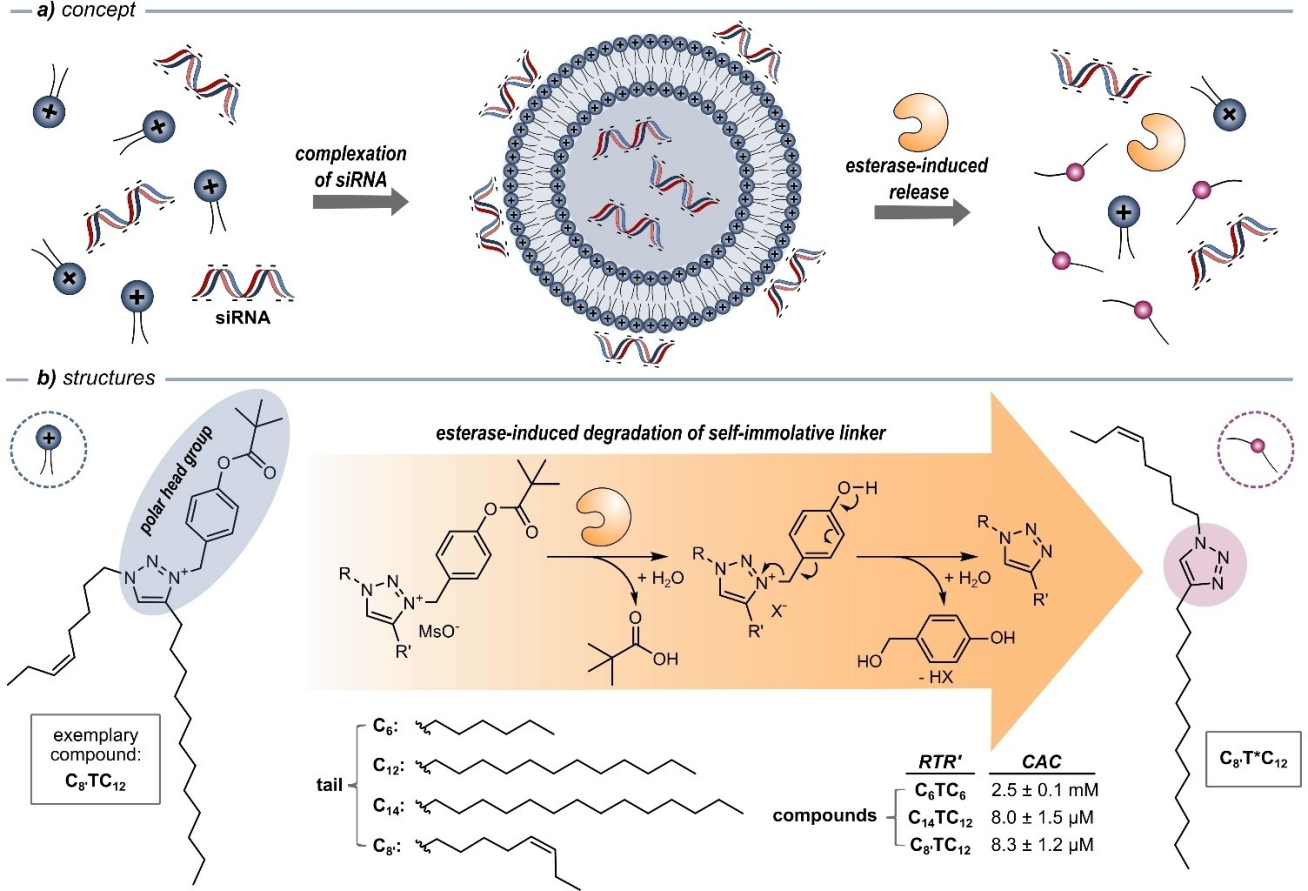
a) Self‐assembly‐based siRNA binding and esterase‐induced siRNA release via cleavage of amphiphiles. b) Structures of triazolium‐based amphiphiles, and proposed cleavage mechanism resulting in the formation of neutral triazoles. Compounds were synthesized with a mesylate (MsO^−^) counterion, but rapid ion exchange occurs in the buffer (X^−^). The critical aggregation concentrations (CAC) were determined using fluorescence spectroscopy with the fluorescent dye 1,6‐diphenyl‐1,3,5‐hexatriene, which shows aggregation‐caused quenching.

A wide range of self‐immolative linkers have been used in stimuli‐responsive drug delivery systems.[^44^, ^50^, ^51^, ^52^] Whereas most known self‐immolative linkers are bound to functional groups such as the hydroxy or amine moiety,[^53^, ^54^, ^55^, ^56^] functionalization of a triazole moiety is rare[^57^, ^58^] and the strategic use of a triazolium group is elusive. We reasoned that the triazole is a promising anchor for self‐immolative groups, because 1) it can be generated in a modular way by CuAAC “click” chemistry, thus enabling the introduction of different (lipid) chains; 2) the conversion of triazoles to triazolium motifs is accompanied with the introduction of a positive charge, enabling subsequent amphiphilic self‐assembly and concomitant complexation of negatively‐charged (bio)molecules such as nucleic acids; 3) upon addition of a certain stimulus, the self‐immolative linker would degrade, leaving the neutral triazole unable to self‐assemble and complex the nucleic acid, thereby triggering its controlled release (Figure 1a).

The first consideration when designing the specific self‐immolative linker was the choice of a stimulus for its cleavage. We decided to use porcine liver esterase as trigger due to its commercial availability and overexpression of esterases in cancer cells.[^59^, ^60^] A benzyl pivalate linker was used (Figure 1b), because its background hydrolysis is negligible in aqueous medium (pH 7.2, phosphate buffered saline (PBS)), while its cleavage has been documented in vitro.^[53]^ Cleavage of the ester bond would result in the release of pivalic acid, followed by a degradation cascade during which quinone methide and the triazole are formed. Quinone methide as a strong Michael acceptor reacts rapidly with water to give 4‐hydroxybenzyl alcohol.

We therefore synthesized three compounds with the same head group, but with varying tails. Overall, our amphiphiles feature a permanent cationic head and two lipophilic chains. We combined tails with different chain lengths (C_6_, C_8_, C_12_ or C_14_) containing either one or no unsaturated bond, resulting in three compounds: **C_6_TC_6_**, **C_8’_TC_12_** and **C_14_TC_12_** (Figure 1b).

To study the aggregation behavior of the compounds, the critical aggregation concentration (CAC) was determined (Figure 1b and Figure S1). Therefore, the fluorescent dye 1,6‐diphenyl‐1,3,5‐hexatriene (DPH) was added to different concentrations of each compound in PBS. This nonpolar dye shows aggregation‐caused quenching in aqueous media, while its photoluminescence increases in a non‐linear way, if the dye is incorporated into a lipophilic nanoenvironment. The CAC of the compound containing the shortest hydrophobic tails (**C_6_TC_6_**) exhibited the highest CAC (2.5±0.1 mM), which we deemed too high for the purposes of drug delivery. Due to their longer hydrophobic tails, compounds **C_8’_TC_12_** and **C_14_TC_12_** did however show CACs three orders of magnitude lower (8.3±1.2 μM and 8.0±1.5 μM, respectively), which is why we pursued these two compounds further. The addition of siRNA (N/P=10, see below) did not have a pronounced effect on the CAC (Table S1).

Dynamic light scattering (DLS) was performed to obtain the average size of the aggregates, and ζ‐potential measurements indicated on the surface charge of those nanoassemblies. Solutions of the compounds in PBS at concentrations higher than the CAC (30 μM) revealed nanoassemblies with a hydrodynamic diameter of 173±25 and 185±5 nm and ζ‐potentials of 35.0±5.6 and 33.4±3.3 mV, for **C_8’_TC_12_** and **C_14_TC_12_**, respectively.

The aggregation of compounds **C_8’_TC_12_** (210 μM) and **C_14_TC_12_** (300 μM) into spherical assemblies with diameters of 140–280 nm (**C_8’_TC_12_**; presumably liposomes) and 160–375 nm (**C_14_TC_12_**) was confirmed by transmission electron microscopy (TEM) (Figure 3a and Figure S2). Confocal fluorescence microscopy using a lipophilic styryl membrane dye showed, at concentrations of 300 μM in Dulbecco's Modified Eagle Medium (DMEM), the formation of spherical aggregates of average diameter around 900 nm for **C_8’_TC_12_** and somewhat larger, less well‐defined aggregates for **C_14_TC_12_** (Figure S3). Further, **C_14_TC_12_** showed less ability to be stained compared to **C_8’_TC_12_**. It needs to be noted that the average size of the nanocarriers lies below the detection limit of optical microscopy and therefore the consistent DLS and TEM data are more reliable regarding (average) size.

### Complexation of siRNA

**Figure 2:**
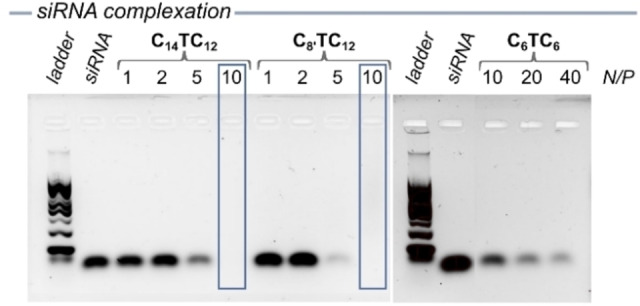
Gel electrophoresis of siRNA complexation by **C_14_TC_12_** (left), **C_8’_TC_12_** (middle) and **C_6_TC_6_** (right) in the presence of siRNA (0.5 μM, N/P=1, 2, 5, 10, 20 and 40). The blue box highlights the runs with full complexation.

The ability of the nanoassemblies of amphiphilic triazolium compounds to complex siRNA was assessed by gel electrophoresis. Each compound was mixed with a fixed amount of siRNA (0.5 μM) at different N/P ratios (molar ratio of positively‐charged triazolium nitrogen atoms per negatively‐charged phosphodiester in siRNA). The final concentration of the triazolium compounds ranged from 21 to 210 μM for **C_8’_TC_12_** and **C_14_TC_12_** (above the CAC) and from 210 to 840 μM for **C_6_TC_6_** (below the CAC). Both **C_8’_TC_12_** and **C_14_TC_12_** showed partial complexation of siRNA at N/P=5 and a complete complexation was found at N/P=10 (Figure 2). On the other hand, **C_6_TC_6_** only showed partial complexation of siRNA up to N/P=40 (Figure 2). In this case the concentration of the compound is below the CAC, thus no assemblies are formed. From this result which demonstrated siRNA complexation by the positively‐charge nanoassemblies of the amphiphilic triazoliums, we selected **C_8’_TC_12_** and **C_14_TC_12_** as the best compounds to move forward.

DLS and ζ‐potential analyses showed nanoassemblies formed at N/P=10 with hydrodynamic diameters of 182±36 and 184±25 nm, and ζ‐potentials of 34.3±4.7 and 25.3±4.2 mV, for **C_8’_TC_12_** and **C_14_TC_12_** (see below), thereby indicating that siRNA complexation has little effect on the size and surface charge of the nanoassemblies, suggesting that the load preferentially fills their inner volume which is predetermined by the amphiphilic self‐assembly of the triazolium compounds. In support of this conclusion, the determination of the CAC in presence of siRNA showed no changes (Figure S1), confirming the absence of any templating role of the siRNA in this case.

**Figure 3:**
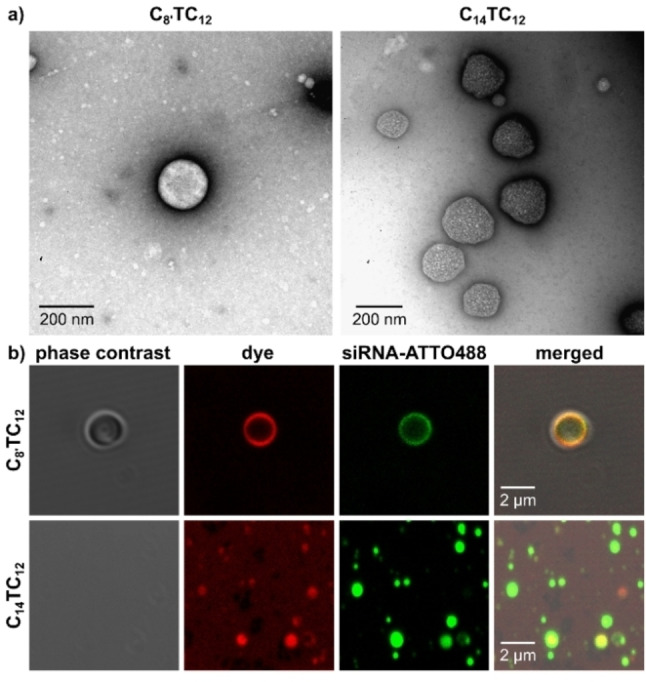
a) Transmission electron microscopy (TEM) images of **C_8’_TC_12_** (210 μM) and **C_14_TC_12_** (300 μM) after negative staining with UO_2_(OAc)_2_. b) Microscopy imaging of **C_8’_TC_12_** and **C_14_TC_12_** (300 μM) complexed with 0.1 μM siRNA‐ATTO488, then stained with lipophilic styryl dye.

The morphologies of the siRNA complexes were visualized by confocal fluorescence microscopy using a fluorophore‐labeled siRNA (Figure 3b). No significant difference in size was observed when the **C_8’_TC_12_** aggregates were treated with labelled siRNA. Striking differences in the complexation mode of siRNA were detected, however, between **C_8’_TC_12_** and **C_14_TC_12_**, with superficial binding dominating with **C_8’_TC_12_** (suggesting liposome structure) and internal binding occurring with **C_14_TC_12_** (suggesting nanoparticle structure).

### Esterase‐induced disassembly and release of siRNA

**Figure 4:**
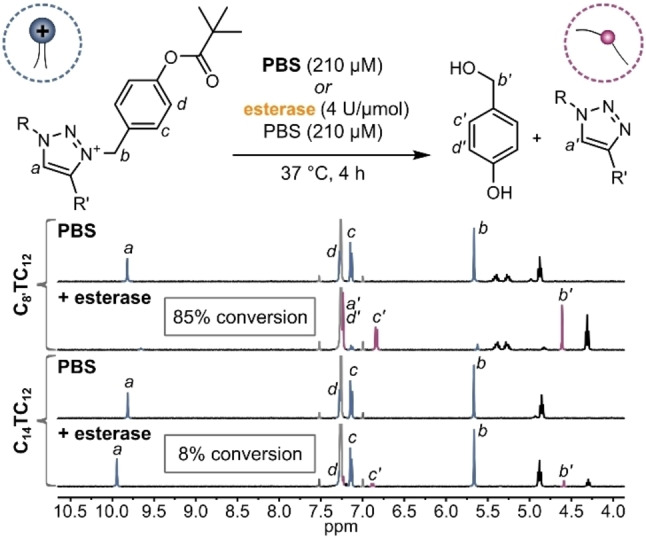
^1^H NMR stacked plot of products after stirring compounds **C_8’_TC_12_** (top) and **C_14_TC_12_** (bottom) at 37 °C for four hours in the absence (upper) or in the presence of esterase (lower).

To study the esterase‐induced conversion of the triazolium to the triazole compounds, we performed the degradation reaction using **C_8’_TC_12_** and **C_14_TC_12_** in the absence and presence of esterase. We chose porcine liver esterase (4 Units per μmol triazolium), PBS buffer (pH 7.2) as solvent, and the temperature was set to 37 °C. After four hours, the organic compounds were extracted from the aqueous phase using ethyl acetate and a ^1^H NMR spectrum was recorded after removal of the solvent (Figure 4). Fortunately, in the absence of esterase no hydrolysis of the ester bond was detected, implying that both triazolium compounds are hydrolytically stable in aqueous medium.

Upon addition of esterase, however, new peaks occurred in the ^1^H NMR spectrum of **C_8’_TC_12_**. The most pronounced shift was observed for the signal belonging to the triazolium/triazole proton from 9.82 (*a*) to 7.23 (*a’*) ppm. Additionally, the signal of the benzyl moiety (*b*) at 5.67 ppm decreased and a new peak at 4.61 ppm arose, which can be attributed to the formation of 4‐hydroxybenzyl alcohol (*b’*). The ratio between triazolium **C_8’_TC_12_** and triazole C_8’_T*C_12_ appears to be 15 : 85 – hence an 85 % conversion of the enzymatic reaction is deduced. It should be noted, that a full conversion is not necessarily needed to realize our release concept, because any concentration of residual triazolium below the CAC will lead to disintegration of the aggregates. When the reaction was performed using **C_14_TC_12_**, only a conversion of around 8 % was observed, even when the reaction was run for 24 h (Figure S4). In light of the kinetic profile observed by NMR spectroscopy, gel electrophoresis and ethidium bromide displacement assay (see below), we propose that the triazole compound C_14_T*C_12_ with longer tails leads to product inhibition. Notably, in all four reactions the signal corresponding to the triazolium proton shows a slight shift compared to the starting material (Figure S21 and Figure S27) due to counterion exchange during the reaction. In all cases, the signal corresponding to the methyl group of the mesylate counterion has disappeared (in essence, MsO^−^ is replaced with a large variety of different anions present in the buffer).

**Figure 5:**
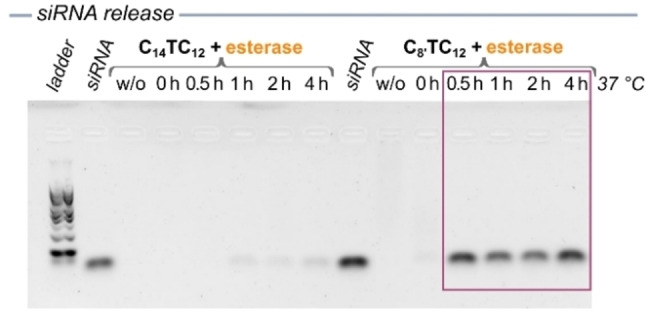
Gel electrophoresis of kinetic monitoring of siRNA release by **C_14_TC_12_** (left) and **C_8’_TC_12_** (right) in the presence of esterase (4 U/μmol) after 0, 0.5, 1, 2 and 4 h (N/P=10). The purple box highlights the runs with complete siRNA release.

The kinetics of esterase‐induced release of the siRNA was also studied by gel electrophoresis. The complexes of **C_8’_TC_12_** and **C_14_TC_12_** with siRNA (N/P=10) were treated with esterase (4 U/μmol) and incubated at 37 °C for different time intervals. After four hours, only a weak siRNA release was observed when using **C_14_TC_12_**. However, in the case of **C_8’_TC_12_** a complete release of siRNA was reached already after 30 minutes (Figure 5). These results are consistent with the data obtained by ^1^H NMR experiments. As explained previously, a complete conversion to the neutral triazole is not crucial for complete siRNA release. The concentration of residual triazolium only needs to decrease below the CAC, while the conversion of the triazolium into triazole lowers the N/P ratio, which also contributes to siRNA release.

**Figure 6:**
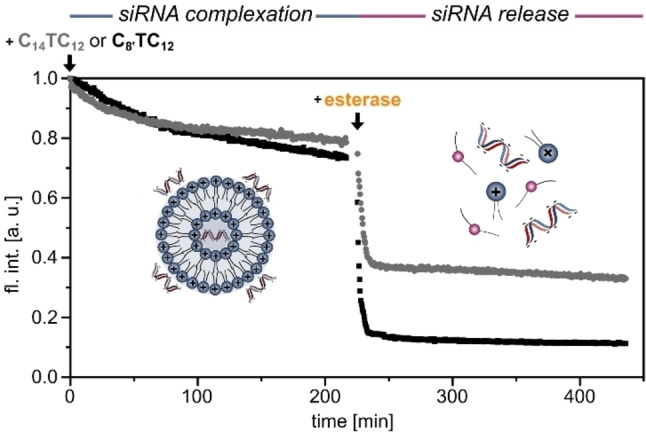
Kinetic monitoring of the fluorescence (590 nm) of an ethidium bromide/siRNA complex (5 μM and 0.5 μM in PBS buffer, respectively) after the addition of compound **C_8’_TC_12_** or **C_14_TC_12_** (210 μM, N/P=10, addition as DMSO solution) at room temperature (T=0 min). After 225 min, the spectrometer was heated to 37 °C and esterase (4 U/μmol) was added to monitor the kinetics of siRNA release.

SiRNA complexation and release were further studied by a fluorescence displacement assay with ethidium bromide (EthBr). The dye shows high fluorescence emission at 590 nm upon intercalation into siRNA. The addition of an siRNA complexing agent leads to a decrease of the emission due to the expulsion of EthBr. A dimethyl sulfoxide (DMSO) solution of compound **C_8’_TC_12_** or **C_14_TC_12_** (21 mM) was added to a solution of siRNA (0.5 μM) and EthBr (5 μM) in PBS to obtain a final concentration of the triazolium compounds of 210 μM (above CAC). A significant decrease of the fluorescence emission intensity was observed over a period of three hours, when the compounds were added as a DMSO solution (Figure 6). When changing the order of addition and omitting DMSO as a solvent, i.e. siRNA was added to a solution of compound **C_8’_TC_12_** or **C_14_TC_12_** and EthBr in PBS, the formation of the complex between the nanoassemblies and siRNA appeared to be much faster (Figure S6). Hence, when DMSO is used as a solvent, the rate of either nanoparticle assembly or siRNA complexation is slowed down. In these assays, we observed no pronounced difference in the complexation ability between compounds **C_8’_TC_12_** and **C_14_TC_12_**, which is in line with the results obtained by gel electrophoresis (see above).

In contrast, differences between both compounds were found when studying siRNA release. Even though the kinetics of siRNA release were almost identical in both reactions (completion after 15 minutes), the outcome was different. The decrease of fluorescence intensity was much more pronounced with **C_8’_TC_12_**. Of note, typically an intensity increase would be expected in such an assay, because the release of siRNA should lead to regeneration of the EthBr/siRNA complex. We propose that our finding of decreased emission upon siRNA release is due to the precipitation of the triazole products C_8’_T*C_12_ and C_14_T*C_12_ which could remove the EthBr dye effectively from the solution (Figure S8). Given the molecular structures, it is reasonable to assume that the flat EthBr dye would co‐precipitate with the triazole compounds. As a control experiment, we measured the fluorescence of the EthBr/siRNA complex in the absence of the amphiphile and found that the intensity was not changing when adding esterase to the complex (Figure S5). Overall, the EthBr assay is valuable as a complement to gel electrophoresis, because it shows in *real time* that siRNA release is completed after 15 to 30 minutes. Such a kinetic profile is of interest, because it matches the process of maturation of endosomes in endocytotic cell penetration.^[61]^

To complete this proof‐of‐principle study, the esterase‐induced transformations were monitored by DLS and ζ‐potential measurements (Table 1). After incubation at 37 °C for four hours, the samples were filtered to remove any precipitate. In the case of **C_8’_TC_12_**, DLS showed a strong decrease in size (hydrodynamic diameter of 2.1±0.4 nm) along with negligible ζ‐potential (−1.03±0.97 mV), which is in agreement with the 85 % enzymatic transformation of the cationic triazolium into the neutral triazole C_8’_T*C_12_ (entry 3, Table 1). The slight negative potential is almost within error and may be explained by the background ζ‐potential of the buffer (Table S3). This result shows, that even without full degradation of the triazolium compounds, the residual amphiphiles are not able to form aggregates. In contrast, when studying **C_14_TC_12_** only minor changes are observed by DLS (entry 7, Table 1), which is in agreement with the low conversion of **C_14_TC_12_** observed by NMR spectroscopy. Crucially, the same trends were found when esterase and siRNA (at N/P=10) were added to the pre‐assembled aggregates, thus confirming that only amphiphile **C_8’_TC_12_** undergoes effective esterase‐induced disassembly and therefore allows the release of siRNA.

**Table 1:** Particle size, polydispersity index (PDI) and ζ‐potential characterization by dynamic light scattering and ζ‐potential measurements of **C_8’_TC_12_** and **C_14_TC_12_** after the addition of siRNA and/or esterase in PBS buffer.

Entry	Compounds	Size±SD [nm]	PDI±SD	ζ‐potential±SD [mV]^[d]^
**1**	**C_8’_TC_12_** ^[a]^	173±25	0.31±0.04	35.0±5.6
**2**	+only siRNA^[b]^	182±36	0.25±0.07	34.3±4.7
**3**	+only esterase^[c]^	2.1±0.4	0.62±0.10	−1.03±0.97
**4**	**+siRNA +esterase** ^[c]^	**2.4±2.9**	**0.67±0.08**	**−2.21±1.93**
**5**	**C_14_TC_12_** ^[a]^	185±5	0.26±0.04	33.4±3.3
**6**	+only siRNA^[b]^	184±25	0.37±0.06	25.3±4.2
**7**	+only esterase^[c]^	241±49	0.35±0.11	26.6±4.5
**8**	**+siRNA +esterase^[c]^**	**189±19**	**0.36±0.01**	**11.3±2.8**

[a] triazolium at concentration of 30 μM. [b] N/P=10, incubation at room temperature for five hours. [c] 4 U/μmol, incubation at 37 °C for four hours. [d] measured in triplicate to rule out error caused by degradation of electrodes by PBS. SD=standard deviation.

## Conclusion

We report the design of a new type of amphiphile that is accessible via CuAAC “click” reaction and features a positively charged triazolium head group capable of the formation of self‐assembled nanocarriers and thereby the complexation of siRNA. At the polar head group, a self‐immolative linker is attached to a triazolium moiety in such a way that addition of esterase leads to the formation of a neutral triazole compound that is unable to form aggregates and therefore releases the siRNA load. Whereas one specific compound (**C_6_TC_6_**) showed a CAC too high to be relevant in drug delivery, two other compounds (**C_8’_TC_12_** and **C_14_TC_12_**) exhibited micromolar CACs and were therefore studied in more detail. While both compounds showed similar siRNA complexation behavior, the siRNA release was much more efficient for compound **C_8’_TC_12_**. We propose that the reason for this finding is that the two long aliphatic chains in the lipophilic product C_14_T*C_12_ lead to product inhibition, for instance via persistent binding to the esterase's active site. Product inhibition on the same magnitude was not observed for compound **C_8’_TC_12_**, in which one aliphatic chain is only roughly half as long (C_8_ vs. C_14_). This delivery system may therefore allow the selective release of siRNA in cells showing a high concentration of esterase, for example in cancer cells. Future work will focus on mixed compositions with additional phospholipids, fine‐tuning of lipophilicity and in vitro studies.

## Conflict of interest

The authors declare no conflict of interest.

1

## Supporting information

As a service to our authors and readers, this journal provides supporting information supplied by the authors. Such materials are peer reviewed and may be re‐organized for online delivery, but are not copy‐edited or typeset. Technical support issues arising from supporting information (other than missing files) should be addressed to the authors.

Supporting Information

## Data Availability

The data that support the findings of this study are available in the supplementary material of this article.
