# Supplementary material for: A Triazolium‐Anchored Self‐Immolative Linker Enables Self‐Assembly‐Driven siRNA Binding and Esterase‐Induced Release
Source: Chemistry. 2022 Dec 16;29(8):e202203311. doi: 10.1002/chem.202203311 (PMC10108132; doi:10.1002/chem.202203311)
Supplement: Supplementary file 1 — Supporting Information [file CHEM-29-0-s001.pdf]

Chemistry–A European Journal

Supporting Information

A Triazolium-Anchored Self-Immolative Linker Enables Self-Assembly-Driven siRNA Binding and Esterase-Induced Release

Selina Hollstein, Lamiaa M. A. Ali, Maëva Coste, Julian Vogel, Nadir Bettache, Sébastien Ulrich,* and Max von Delius*

Supporting Information
©Wiley-VCH 2021
69451 Weinheim, Germany

A triazolium-anchored self-immolative linker enables self-assembly-driven siRNA binding and esterase-induced release

Selina Hollstein,[a] Lamiaa M. A. Ali,[b,c] Maëva Coste,[b] Julian Vogel,[a] Nadir Bettache,[b] Sébastien Ulrich,*[b] and Max von Delius*[a]

DOI: 10.1002/anie.2021XXXXX

Table of Contents

1. General Experimental Section....3
2. Synthesis and Characterization....6
3. Critical Aggregation Concentration....14
4. Transmission Electron Microscopy....15
5. Confocal Microscopy....16
6. Esterase-induced degradation....17
7. Ethidium Bromide Displacement Assay....18
8. Dynamic Light Scattering and ζ-Potential....19
9. NMR Spectra....21

1. General Experimental Section

All commercially available chemicals were purchased from Sigma Aldrich and used without further purification. $CDCl_3$ was stored over molecular sieves (3 Å). Anhydrous solvents were dried prior to use in a MBraun SPS-800 instrument. Porcine liver esterase was used as lipophilized powder (Sigma Aldrich). Dulbecco's phosphate buffered saline without calcium and magnesium (Thermo Fisher) was used.

The siRNA (siCtrl) sequence is: 5'-CGUACGCGGAAUACUUCGAdTdT-3' (sense strand) and 5'-UCGAAGUAUUCCGCGUACG dTdT-3' (anti-sense strand) which was purchased from Eurogentec (Serring, Belgium).

NMR spectra were recorded on Bruker Avance Neo 400 spectrometers (^{1}H: 400 MHz, ^{13}C: 100 MHz) at 293 K. The spectra were calibrated to the residual solvent peaks (^{1}H NMR: 7.26 ppm ($CDCl_3$), ^{13}C NMR: 77.16 ppm ($CDCl_3$). Chemical shifts (δ) are denoted in ppm and coupling constants (J) in Hz.

High resolution mass spectra were recorded on Agilent QTOF 6546 using electrospray ionization (ESI). Acetonitrile was used as solvent.

The normal-phase flash column chromatography was performed using silica 60 with a particle size of 0.04 – 0.063 mm from Macherey-Nagel. The reversed-phase column chromatography was performed with Interchim PuriFlash 430 by using C18-functionalized silica gel with a particle size of 15 µm.

Critical aggregation concentration experiments were performed on a PerkinElmer LS 55 spectrophotometer at 25 °C using either a 1 mL or 3 mL cuvette. A stock solution of 1,6-diphenyl-1,3,5-hexatriene (DPH) in inhibitor-free THF (1 mM) was prepared. Stock solutions of the triazolium compounds were prepared in PBS buffer and diluted to different concentration. After addition of DPH (10 µM), the solutions were kept in the dark overnight prior to the measurements. The excitation wavelength was set to 355 nm.

For gel electrophoresis experiments, a fixed concentration of siCtrl (0.5 µM) was mixed with the appropriate amounts of triazolium compounds in order to reach N/P ratios (molar ratio of positively-charged nitrogens in the triazolium per negatively-charged phosphodiester in siRNA) of 1, 2, 5, 10, 20 and 40 in PBS buffer (20 µL total volume). The solutions were incubated at 25 °C for one hour, and 2 µL 10X BlueJuice^{TM} Gel Loading Buffer (Thermo Fisher Scientific) was added to the mixture. For siRNA release studies, esterase (4 U/µmol) was admixed to solutions with N/P ratio of 10 and samples were incubated at 37 °C for different time intervals (0, 0.5, 1, 2, 4 h). Electrophoresis was carried out on a 2% w/v agarose gel mixed with GelRed^{TM} nucleic acid gel stain (Interchim, France) in 1X TBE buffer (90 mM Tris-borate/2 mM EDTA, pH 8.2). The gel was run in 0.5X TBE at 50 V for 30 minutes. A 100 bp

DNA ladder from Sigma-Aldrich (Saint-Quentin-Fallavier, France) was used as a reference for the gel. The GelRed-stained siRNA was visualized using a TFX-20 M model-UV transilluminator (Vilber Lourmat, Marne-la-Vallée, France).

Dynamic light scattering (DLS) and ζ-potential measurements were performed on a NanoZetasizer (Malvern Instruments) at 25 °C using PVA disposable cuvettes and DTS1070 zeta potential cells. DLS measurements were recorded with a 173° backscatter angle at λ = 633 nm. Solutions of the triazolium compounds in PBS buffer were prepared (30 µM) and mixed with siRNA (70 nM, N/P = 10) and/or esterase (4 U/µmol) with a final volume of 1 mL. The siRNA complexes were incubated at 25 °C for a minimum of one hour. After the addition of esterase, the mixture was incubated at 37 °C for four hours. Each sample was vortexed and filtered through a syringe filter (450 nm) prior to the measurement. Three measurements were made with 10 runs (7 seconds) each, and repeated two times with a different batch to rule out any errors due to electrode degradation caused by PBS buffer.

siRNA release studies by ^{1}H NMR spectroscopy. Stock solutions of triazolium compounds (300 µM) and esterase (100 U/mL) in PBS were prepared. Triazolium stock solutions were diluted with PBS to a final concentration of 210 µM. Esterase (4 U/µmol) was added and the suspension was left stirring at 37 °C for 4 hours. The aqueous solution was extracted with EtOAc. After evaporation of the solvent, a ^{1}H NMR spectrum was recorded.

Ethidium bromide displacement assay was performed using a Xenius spectrofluorimeter (Safas, France) and 3 mL Quartz cuvettes with stopper. Excitation wavelength was set to 546 nm and emission was measured at 590 nm at room temperature. In method 1, the spectrofluorimeter was set to 37 °C prior to the addition of esterase. After each addition, the cuvette was shaken to ensure complete mixing. Stock solutions of siRNA (100 µM in RNase free water), EthBr (2.5 mM) and esterase (0.25 U/µL) in PBS were prepared. Stock solutions of triazolium compounds were prepared either in DMSO (21 mM) or in PBS buffer (**$C_{14}TC_{12}$**: 300 µM, **$C_{8'}TC_{12}$**: 500 µM).

Method 1: 2 µL EthBr solution and 5 µL siRNA were diluted with PBS to obtain final concentrations of 5 µM and 0.5 µM, respectively. After an equilibration time of 10 minutes, the DMSO solution of the triazolium compounds were added to obtain a final concentration of 210 µM. After 225 minutes, esterase was added (4 U/µmol).

Method 2: 2 µL EthBr solution and the PBS solution of the triazolium compounds were diluted to obtain final concentrations of 5 µM and 210 µM, respectively. After an equilibration time of 10 minutes, 5 µL siRNA was added (final concentration of 0.5 µM). The first fluorescence value was recorded after 1 minute. After 36 minutes, esterase (67 U/µmol) was added and the fluorescence was measured at room temperature.

Confocal microscopy imaging. Stock solutions of triazolium compounds **C_{8}'TC_{12}** and **$C_{14}TC_{12}$** were freshly prepared in DMEM at a concentration of 2.5 mM and 1.5 mM, respectively, followed by a sonication for 10 min. For imaging the siRNA complexes, they were mixed with 100 nM of siRNA-ATTO488 (sense: 5-CGUACGCGGAAUACUUCGA55-3′ and anti-sense: 5′-UCGAAGUAUUCCGCGUACG55-3′) at a final concentration of 300 µM and incubated for 1 h at 25 °C. After the incubation period, solutions were mixed with FM™ 4-64 Dye (Invitrogen, USA) at a final concentration of 5 µg mL^{-1}. After 10 min, few drops of the solution were added on a slide and covered with a coverslip. Imaging was performed using confocal microscopy LSM880 (Carl Zeiss, France). Imaging parameters for the FM™ 4-64 dye and siRNA-ATTO488 were excitation at 561 nm and 488 nm and emission at 599-723 nm and 502-585 nm, respectively, using a high magnification (63x/1.4 OIL Plan-Apo). Phase contrast images and (15X) zoom of the interested area were performed.

List of abbreviations

CAC	critical aggregation concentration	MeCN	acetonitrile
DCM	dichloromethane	MeOH	methanol
DLS	dynamic light scattering	MsCl	mesyl chloride
DMEM	Dulbecco's Modified Eagle's Medium	OMs	mesylate
DMSO	dimethyl sulfoxide	PBS	phosphate buffered saline
DMF	*N*,*N*-dimethylformamide	PDI	polydispersion index
DPH	1,6-diphenyl-1,3,5-hexatriene	r.t.	room temperature
ESI	electronspray ionization	SD	standard deviation
EthBr	ethidium bromide	TBTA	Tris((1-benzyl-4-triazolyl)methyl)amine
EtOAc	ethyl acetate	TEA	triethylamine
equiv.	equivalents	THF	tetrahydrofuran
HRMS	high resolution mass spectrometry		

2. Synthesis and Characterization

Overview of the synthesis of compounds ***C₆TC₆*****,** ***C₁₄TC₁₂*** *and* ***C₈'TC₁₂***

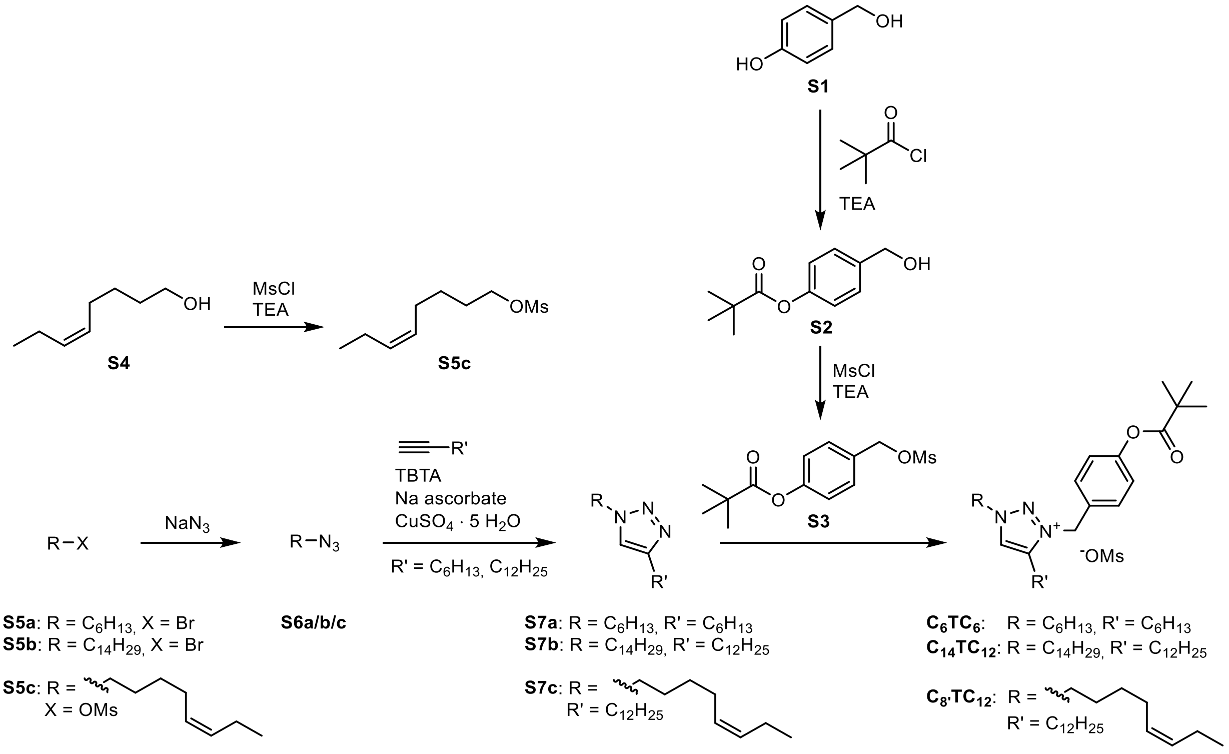

Synthesis of compound (S2)

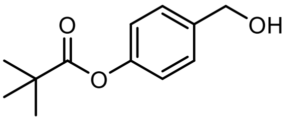

4-Hydroxybenzyl alcohol **S1** (4.00 g, 32.2 mmol, 1 equiv.) was suspended in 250 mL anhydrous DCM under argon and TEA (8.95 mL, 6.50 g, 64.4 mmol, 2 equiv.) was added dropwise. After cooling the suspension to 0 °C, trimethylacetyl chloride (4.23 mL, 4.24 g, 35.4 mmol, 1.1 equiv.) was added dropwise. The reaction mixture was allowed to warm up to room temperature and stirred overnight. The reaction mixture was partitioned between 100 mL DCM and 200 mL water, and the organic layer was washed with water (3 x 50 mL) and brine (50 mL). The organic layer was dried over $MgSO_4$ and the organic solvent was removed under reduced pressure. The crude product was purified by column chromatography (EtOAc/PE, 4:6) to obtain the clean product as a colourless solid (4.25 g, 13.8 mmol, 43%).

^{1}H NMR (400 MHz, $CDCl_3$): δ 7.38 (d, *J* = 8.6 Hz, 2H, CH *Ar*), 7.05 (d, *J* = 8.5 Hz, 2H, CH *Ar*), 4.69 (d, *J* = 9.2 Hz, 2H, CH_2 *benzylic*), 1.63 (t, *J* = 6.0 Hz, 1H, *OH*), 1.36 (s, 9H, CH_3 t-*Bu*).

^{13}C NMR {1H} (101 MHz, $CDCl_3$): δ 177.3, 150.7, 138.4, 128.1, 121.7, 64.9, 39.2, 27.3.

Synthesis of compound (S3)

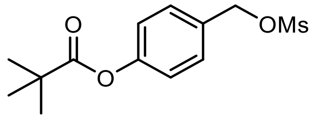

S2 (100 mg, 480 µmol, 1 equiv.) was dissolved in 12 mL anhydrous DCM under argon. TEA (201 µL, 146 mg, 1.44 mmol, 3 equiv.) was added dropwise to the solution. After cooling to -10 °C, MsCl (93.6 µL, 138 mg, 1.20 mmol, 2.5 equiv.) was added during 15 min and the reaction mixture was stirred for further 3 hours. 20 mL cooled DCM was added and the organic layer was washed with cooled sat. aqueous $NaHCO_3$ solution (3 x 40 mL), cooled water (3 x 40 mL) and cooled brine (40 mL). The organic layer was dried over $MgSO_4$ and the solvent was removed under vacuum. The crude product was used without further purification (134 mg, 467 µmol, 97%).

^{1}H NMR (400 MHz, $CDCl_3$): δ 7.43 (d, *J* = 8.5 Hz, 2H, CH *Ar*), 7.10 (d, *J* = 8.5 Hz, 2H, CH *Ar*), 5.22 (s, 2H, CH_2 *benzylic*), 2.91 (s, 3H, CH_3 O*Ms*), 1.35 (s, 9H, CH_3 t-*Bu*).

^{13}C NMR {1H} (101 MHz, $CDCl_3$): δ 177.0, 152.0, 130.9, 130.2, 122.2, 70.9, 39.2, 38.5, 27.2.

Synthesis of compound (S7a)

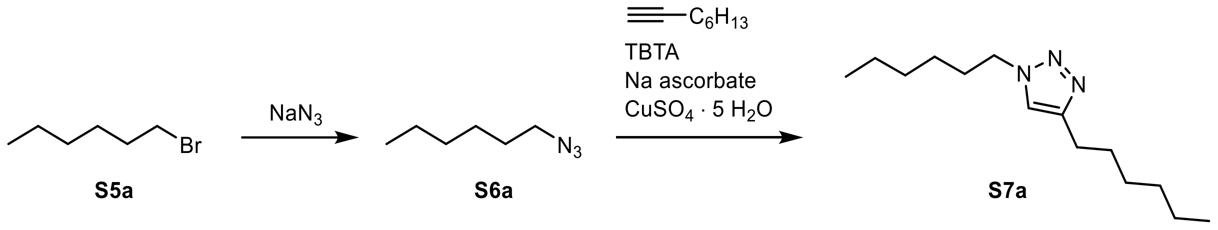

1-Bromohexane (**S5a**) (1.00 mL, 1.19 g, 7.21 mmol, 1 equiv.) was dissolved in 30 mL anhydrous DMF under argon. Sodium azide (938 mg, 14.4 mmol, 2 equiv.) was added, the solution was heated to 80 °C and left stirring for 18 hours. After cooling to room temperature, 50 mL DCM and 50 mL water were added. The aqueous layer was extracted with DCM (2 x 50 mL) and the combined organic layers were washed with water and brine. The combined aqueous layers were treated with iodine and 10 wt% NaS_2O_3 solution to quench residual azide. The organic layer was dried over $MgSO_4$, and DCM was removed using a rotary evaporator, resulting in a DMF solution of the crude product (**S6a**). To this solution, 10 mL water and 20 mL THF were added. 1-Octyne (1.06 mL, 793 mg, 7.21 mmol, 1 equiv.) and TBTA (37.8 mg, 71.2 µmol, 0.01 equiv.) were dissolved in the solvent mixture. After degassing the solution for 20 min at 60 °C, sodium ascorbate (28.2 mg, 142 µmol, 0.02 equiv.) and copper(II) sulfate pentahydrate (17.8 mg, 71.2 µmol, 0.01 equiv.) were added. After stirring at 60 °C under argon for 18 hours, THF was removed under vacuum and the aqueous layer was extracted with ethyl acetate. The combined organic layers were washed with water and brine, and dried over $MgSO_4$. After removal of the solvent under vacuum, the crude product was purified by column chromatography (DCM/MeOH, 99:1). The clean product was yielded as a colourless solid (1.33 g, 5.61 mmol, 78%).

^{1}H NMR (400 MHz, $CDCl_3$) δ 7.23 (s, 1H, CH *triazole*), 4.29 (t, *J* = 7.2 Hz, 2H, CH_2 *alkyl*), 2.69 (t, *J* = 7.7 Hz, 2H, CH_2 *alkyl*), 1.92 – 1.78 (m, 2H, CH_2 *alkyl*), 1.72 – 1.57 (m, 2H, CH_2 *alkyl*), 1.40 – 1.22 (m, 12H, CH_2 *alkyl*), 0.92 – 0.82 (m, 6H, CH_3 *alkyl*).

^{13}C NMR {1H} (101 MHz, $CDCl_3$): δ 148.5, 120.4, 50.3, 31.7, 31.3, 30.4, 29.6, 29.0, 26.3, 25.8, 22.7, 22.5, 14.2, 14.0.

HR-ESI-MS: m/z = 238.2278 $[M+H]^+$ (calculated: m/z = 238.2283).

Synthesis of compound (C_6TC_6)

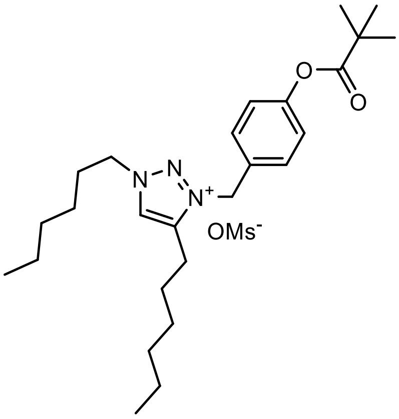

Compound (**S7a**) (100 mg, 0.422 mmol, 1 equiv.) and freshly prepared compound (**S3**) (605 mg, 2.11 mmol, 5 equiv.) were dissolved in 10 mL anhydrous acetonitrile. The solution was stirred for one day at 90 °C under argon. The solvent was removed under reduced pressure and the crude product was purified by flash column chromatography (DCM/MeOH, 9:1). Residual impurities were removed using reversed phase column (MeCN/water, 3:7 to 1:1). The solvent was removed by lyophilization and the clean product was obtained as a colourless solid (122 mg, 0.233 mmol, 55%).

^{1}H NMR (400 MHz, $CDCl_3$) δ 9.10 (s, 1H, CH *triazolium*), 7.32 – 7.27 (m, 2H, CH *Ar*), 7.15 – 7.08 (m, 2H, CH_2 *benzylic*), 5.68 (s, 2H, CH_2 *alkyl*), 4.72 (t, *J* = 7.4 Hz, 2H, CH_2 *alkyl*), 2.78 (t, *J* = 7.9 Hz, 2H, CH_2 *alkyl*), 2.72 (s, 3H, CH_3 *OMs*), 2.02 (p, *J* = 7.4 Hz, 2H, CH_2 *alkyl*), 1.63 (p, *J* = 7.7 Hz, 2H, CH_2 *alkyl*), 1.41 – 1.18 (m, 21H, CH_2 *alkyl* + CH_3 t-*Bu*), 0.91 – 0.79 (m, 6H, CH_3 *alkyl*).

^{13}C NMR {1H} (101 MHz, $CDCl_3$): δ 176.9, 152.3, 144.7, 130.4, 129.3, 128.5, 123.0, 54.4, 54.3, 39.6, 39.3, 31.3, 31.1, 29.5, 28.9, 27.3, 27.2, 26.0, 23.8, 22.5, 22.5, 14.1, 14.0.

HR-ESI-MS: m/z = 428.3279 $[M\text{-}OMs]^+$ (calculated: m/z = 428.3277).

Synthesis of compound (S6b)

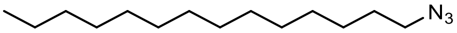

Compound (**S6b**) was synthesized according to the same procedure as compound (**6a**). All solvents were removed under vacuum and the isolated product was yielded as a yellow solid (3.39 g, 14.2 mmol, 87%).

^{1}H NMR (400 MHz, $CDCl_3$): 3.25 (t, *J* = 7.0 Hz, 2H, CH_2 *alkyl*), 1.68 – 1.51 (m, 2H, CH_2 *alkyl*), 1.43 – 1.19 (m, 22H, CH_2 *alkyl*), 0.92 – 0.84 (m, 3H, CH_3 *alkyl*).

^{13}C NMR {1H} (101 MHz, $CDCl_3$): δ 51.7, 32.1, 29.8, 29.8, 29.8, 29.8, 29.7, 29.6, 29.5, 29.3, 29.0, 26.9, 22.8, 14.3.

Synthesis of compound (S7b)

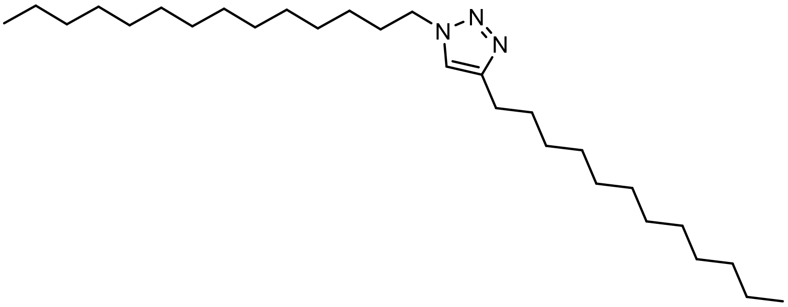

Compound (**S3b**) (1.60 g, 6.69 mmol, 1 equiv.), TBTA (35.5 mg, 66.9 µmol, 0.01 equiv.) and 1-tetradecyne, (1.68 mL, 1.33 g, 6.69 mmol, 1 equiv.) were dissolved in a solvent mixture of water/THF/*t*-BuOH (1:2:1, 40 mL). After degassing the solution for 20 min at 50 °C, sodium ascorbate (26.6 mg, 134 µmol, 0.02 equiv.) and copper(II) sulfate pentahydrate (16.7 mg, 66.9 µmol, 0.01 equiv.) were added to the solution. After stirring at 50 °C under argon for three days, the crude product precipitated as a colourless solid. The precipitate was washed with water and ethyl acetate. To the residual reaction mixture, water was added and the aqueous layer was extracted with ethyl acetate. The combined organic layers were dried over $MgSO_4$ and the solvent was removed under reduced pressure. The crude product was purified by column chromatography (DCM/MeOH, 98:2). The clean product was yielded as a yellow solid (1.75 g, 4.03 mmol, 60%).

^{1}H NMR (400 MHz, CD_2Cl_2): δ 7.29 (s, 1H, CH *triazole*), 4.27 (t, *J* = 7.2 Hz, 2H, CH_2 *alkyl*), 2.79 – 2.48 (m, 2H, CH_2 *alkyl*), 1.93 – 1.77 (m, 2H, CH_2 *alkyl*), 1.69 – 1.58 (m, 2H, CH_2 *alkyl*), 1.42 – 1.17 (m, 40H, CH_2 *alkyl*), 0.93 – 0.73 (m, 6H, CH_3 *alkyl*).

^{13}C NMR {1H} (101 MHz, $CDCl_3$): δ 148.6, 120.4, 50.3, 32.1, 30.5, 29.8, 29.8, 29.7, 29.7, 29.7, 29.5, 29.5, 29.4, 29.2, 26.7, 25.9, 22.8, 14.3.

HR-ESI-MS: m/z = 434.4485 $[M+H]^+$ (calculated: m/z = 434.4474).

Synthesis of compound ($C_{14}TC_{12}$)

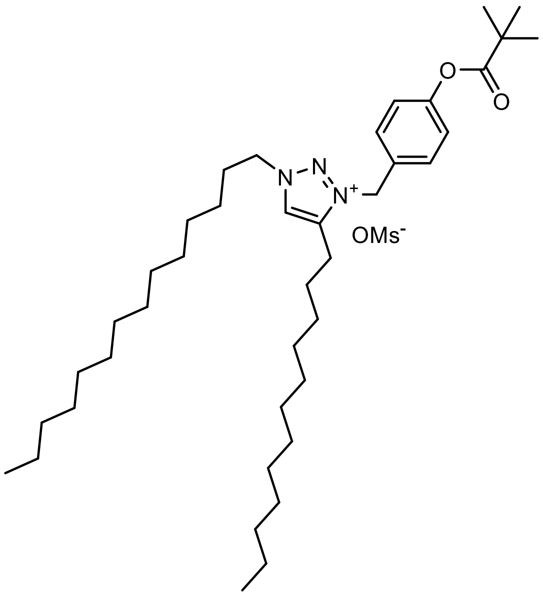

Compound (**$C_{14}TC_{12}$**) was synthesized according to the same procedure as compound (**C_6TC_6**). The crude product was purified by flash column chromatography (DCM/MeOH, 9:1). Residual impurities were removed using reversed phase column chromatography (MeCN/water, 6:4 to 1:0). The clean product was yielded as a yellow oil (178 mg, 250 µmol, 36%).

^{1}H NMR (400 MHz, $CDCl_3$): δ 9.12 (s, 1H, CH *triazolium*), 7.31 – 7.27 (m, 2H, CH *Ar*), 7.13 (d, *J* = 8.6 Hz, 2H, CH *Ar*), 5.64 (s, 2H, CH_2 *benzylic*), 4.74 (t, *J* = 7.4 Hz, 2H, CH_2 *alkyl*), 2.81 – 2.75 (m, 2H, CH_2 *alkyl*), 2.74 (s, 3H, CH_3 *OMs*), 2.13 – 1.96 (m, 2H, CH_2 *alkyl*), 1.74 – 1.59 (m, 2H, CH_2 *alkyl*), 1.44 – 1.18 (m, 49H, CH_2 *alkyl* + CH_3 t-*Bu*), 0.93 – 0.79 (m, 6H, CH_3 *alkyl*).

^{13}C NMR {1H} (101 MHz, $CDCl_3$): δ 176.9, 152.3, 144.7, 130.3, 129.3, 128.5, 123.0, 54.4, 54.3, 39.6, 39.3, 32.0, 29.8, 29.8, 29.8, 29.7, 29.7, 29.7, 29.6, 29.5, 29.5, 29.5, 29.5, 29.5, 29.2, 29.1, 29.0, 27.4, 27.2, 26.3, 23.8, 22.8, 14.2.

HR-ESI-MS: m/z = 624.5482 $[M\text{-}OMs]^+$ (calculated: m/z = 624.5468), 1344.0723 $[2M\text{-}OMs]^+$ (calculated: 1344.0739).

Synthesis of compound (S5c)

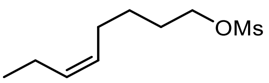

Cis-5-octene-1-ol (**S4**) (1.00 mL, 849 mg, 6.63 mmol, 1 equiv.) was dissolved in 30 mL anhydrous DCM under argon. TEA (2.77 mL, 2.01 g, 19.9 mmol, 3 equiv.) was added dropwise. After cooling the solution to -10 °C with an ice bath containing NaCl, MsCl (1.29 mL, 1.91 g, 16.6 mmol, 2.5 equiv.) was added within 15 min. After stirring for 3 hours, 30 mL cooled DCM was added and the organic layer was washed with cooled saturated aq. $NaHCO_3$ solution (50 mL), cooled water (50 mL) and cooled brine (50 mL). The organic phase was dried over $MgSO_4$ and the product was obtained after removing the solvent under reduced pressure as a yellow oil. The crude product was used without further purification (1.37 g, 6.63 mmol, quant.).

^{1}H NMR (400 MHz, $CDCl_3$): δ 5.40 – 5.30 (m, 1H, CH *vinylic*), 5.27 (s, 1H, CH *vinylic*), 4.16 (t, *J* = 6.5 Hz, 2H, CH_2 *OMs*), 2.94 (s, 3H, CH_3 *OMs*), 2.07 – 1.90 (m, 4H, CH_2 *alkyl*), 1.75 – 1.64 (m, 2H, CH_2 *alkyl*), 1.48 – 1.36 (m, 2H, CH_2 *alkyl*), 0.90 (t, *J* = 7.5 Hz, 3H, CH_3 *alkyl*).

^{13}C NMR {1H} (101 MHz, $CDCl_3$): δ 132.4, 128.0, 70.1, 37.2, 28.5, 26.3, 25.4, 20.4, 14.2.

Synthesis of compound (S7c)

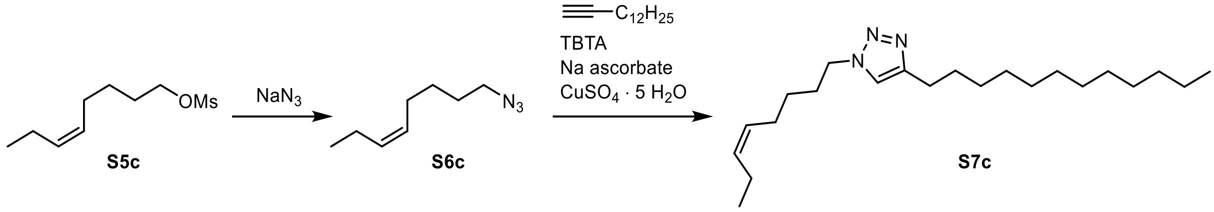

Compound (**S7c**) was synthesized according to the procedure for compound (**S7a**) with some deviation. After completion of the reaction, the solvent was removed and the residual crude product was dissolved in hot EtOAc. The resulting suspension was filtered while still hot. The residual organic layer was washed with water and brine, dried over $MgSO_4$ and the solvent was removed under vacuum. The crude product was purified by column chromatography (DCM/MeOH, 98:2). The clean product was yielded as a colourless solid (1.06 g, 3.05 mmol, 46%).

^{1}H NMR (400 MHz, $CDCl_3$): δ 7.23 (s, 1H, CH *triazole*), 5.51 – 5.33 (m, 1H, CH *vinylic*), 5.33 – 5.21 (m, 1H, CH *vinylic*), 4.31 (t, *J* = 7.2 Hz, 2H, CH_2 *alkyl*), 2.70 (t, *J* = 7.7 Hz, 2H, CH_2 *alkyl*), 2.20 – 1.95 (m, 4H, CH_2 *alkyl*), 1.89 (dq, *J* = 10.0, 7.3 Hz, 2H, CH_2 *alkyl*), 1.66 (p, *J* = 7.5 Hz, 2H, CH_2 *alkyl*), 1.46 – 1.20 (m, 20H, CH_2 *alkyl*), 0.95 (t, *J* = 7.5 Hz, 3H, CH_3 *alkyl*), 0.88 (t, *J* = 6.8 Hz, 3H, CH_3 *alkyl*).

^{13}C NMR {1H} (101 MHz, CDCl$_3$): δ 148.6, 132.7, 128.1, 120.4, 50.2, 32.1, 30.0, 29.8, 29.8, 29.7, 29.7, 29.5, 29.5, 29.4, 26.7, 26.5, 25.9, 22.8, 20.7, 14.5, 14.3.

HR-ESI-MS: m/z = 348.3385 [M+H]$^+$ (calculated: m/z = 348.3379).

Synthesis of compound ($C_{8'}TC_{12}$)

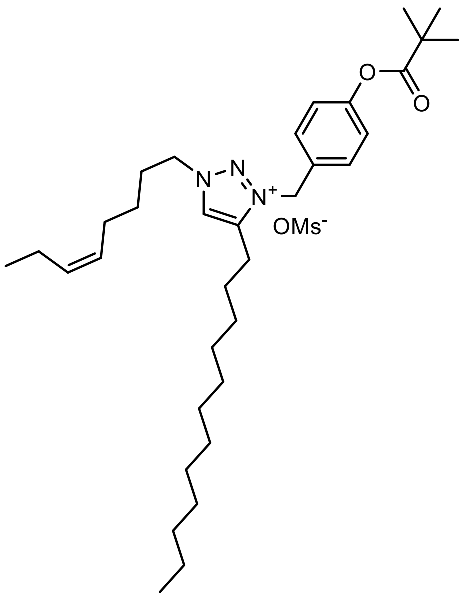

Compound (**$C_{8'}TC_{12}$**) was synthesized according to the same procedure as compound (**C_6TC_6**). The crude product was purified by flash column chromatography (DCM/MeOH, 9:1). Residual impurities were removed using reversed phase column chromatography (MeCN/water, 1:1 to 1:0). The solvent was removed by lyophilization and the clean product was obtained as a colourless solid (322 mg, 508 µmol, 35%).

^{1}H NMR (400 MHz, CDCl$_3$): δ 9.16 (s, 1H, CH *triazolium*), 7.31 – 7.26 (m, 2H, CH *Ar*), 7.16 – 7.09 (m, 2H, CH *Ar*), 5.64 (s, 2H, CH$_2$ *benzylic*), 5.48 – 5.36 (m, 1H, CH *vinylic*), 5.31 – 5.21 (m, 1H, CH *vinylic*), 4.77 (t, *J* = 7.3 Hz, 2H, CH$_2$ *alkyl*), 2.82 – 2.75 (m, 2H, CH$_2$ *alkyl*), 2.74 (s, 3H, CH$_3$ *OMs*), 2.14 – 1.97 (m, 6H, CH$_2$ *alkyl*), 1.73 – 1.64 (m, 2H, CH$_2$ *alkyl*), 1.43 (p, *J* = 7.4 Hz, 2H, CH$_2$ *alkyl*), 1.35 (s, 9H, CH$_3$ t-*Bu*), 1.32 – 1.19 (m, 18H, CH$_2$ *alkyl*), 0.95 (t, *J* = 7.5 Hz, 3H, CH$_3$ *alkyl*), 0.88 (t, *J* = 6.8 Hz, 2H, CH$_3$ *alkyl*).

^{13}C NMR {1H} (101 MHz, CDCl$_3$): δ 152.4, 144.7, 133.0, 130.5, 129.3, 128.4, 127.7, 123.1, 54.4, 54.3, 39.6, 39.3, 32.1, 29.8, 29.7, 29.6, 29.5, 29.3, 29.2, 29.0, 27.4, 27.2, 26.4, 26.4, 23.9, 22.8, 20.7, 14.5, 14.3.

HR-ESI-MS: m/z = 538.4378 [M-OMs]$^+$ (calculated: m/z = 538.4373).

3. Critical Aggregation Concentration

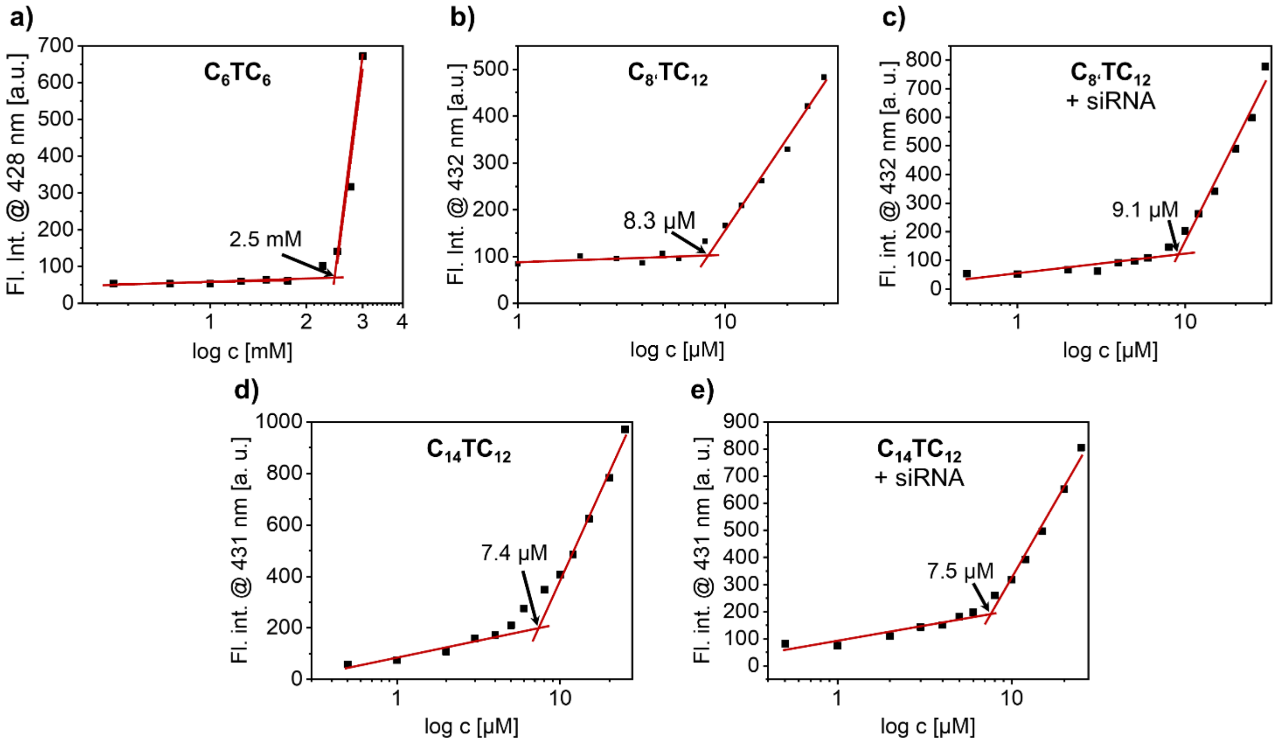

Figure S1: The CAC of the triazolium compounds **C_6TC_6**, **$C_{8'}TC_{12}$** and **$C_{14}TC_{12}$** in the absence or presence of siRNA (N/P = 10) were determined by monitoring the fluorescence intensity of DPH dye (10 µM) at 355 nm in a dilution series of amphiphiles. Upon aggregation, the dye is incorporated into the hydrophobic part of the amphiphile and therefore, the fluorescence is increased. Values for the CAC in the absence of siRNA were measured in triplicate and representative single measurements are shown.

Table S1: Summary of critical aggregation concentrations of triazolium compounds in the absence or presence of siRNA. Values for the CAC in the absence of siRNA were measured in triplicate.

siRNA	C_6TC_6	$C_{8'}TC_{12}$	$C_{14}TC_{12}$
w/o	2.5 ± 0.1 mM	8.3 ± 1.2 µM	8.0 ± 1.5 µM
w/		9.1 µM	7.5 µM

4. Transmission Electron Microscopy

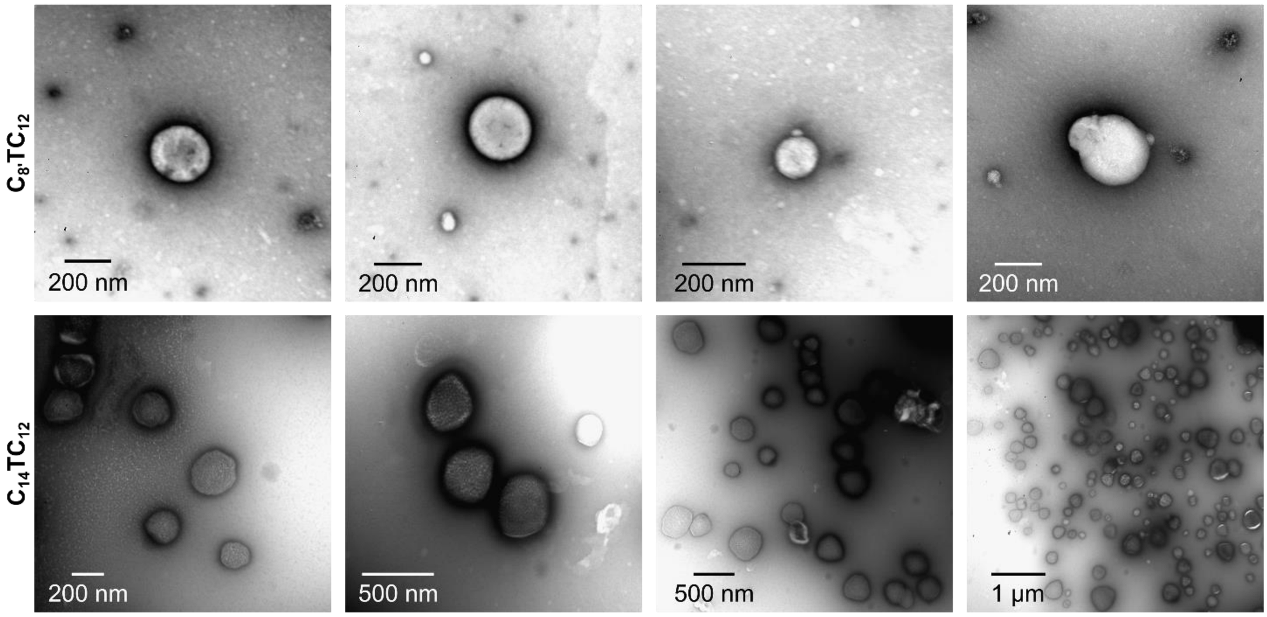

Figure S2: Transmission electron microscopy (TEM) images of $\mathbf{C_{8'}TC_{12}}$ (210 µM) and $\mathbf{C_{14}TC_{12}}$ (300 µM) after negative staining with $UO_2(OAc)_2$ showing spherical nanoassemblies with diameters of 140-280 nm ($\mathbf{C_{8'}TC_{12}}$) and 160-375 nm ($\mathbf{C_{14}TC_{12}}$).

5. Confocal Microscopy

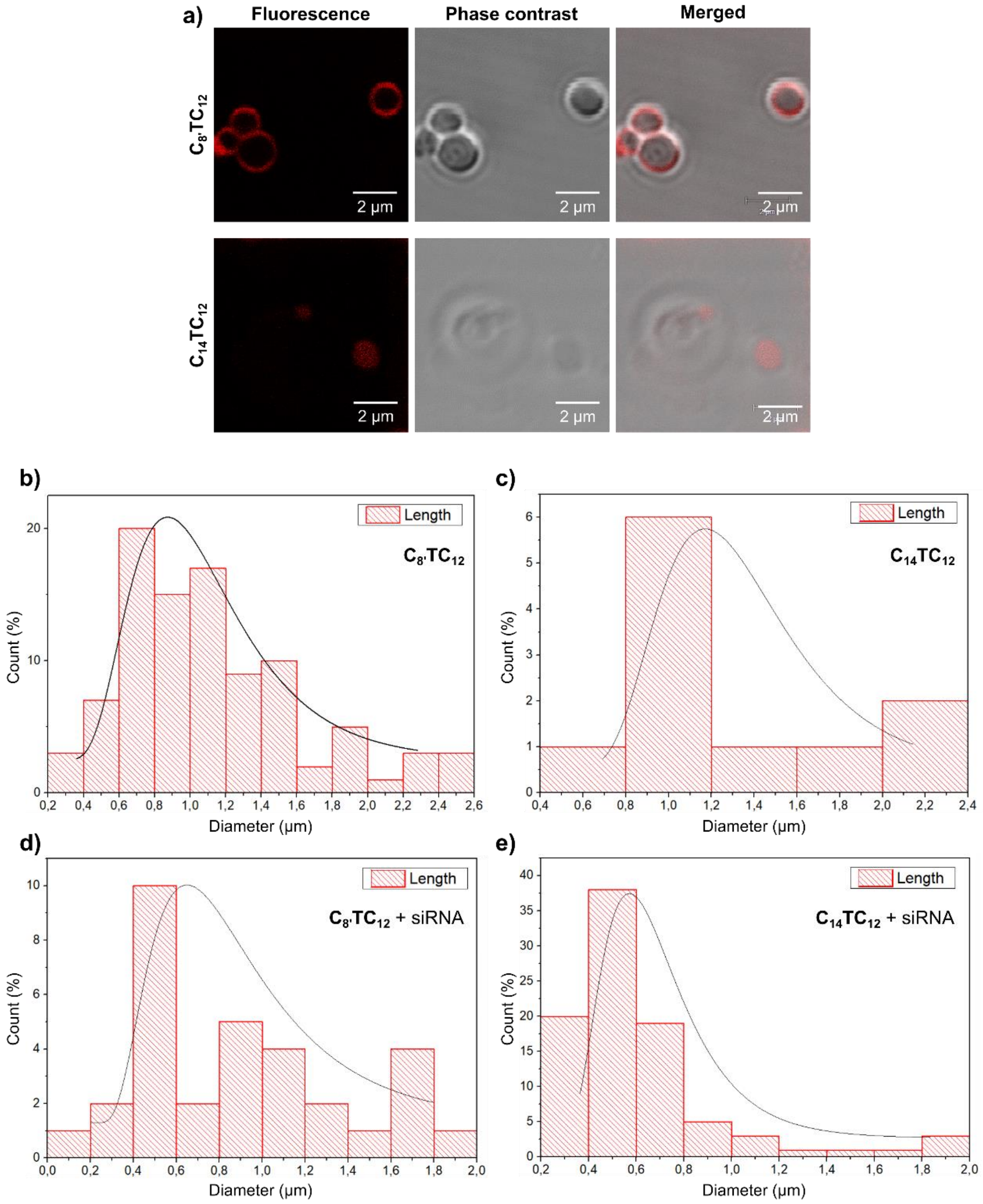

Figure S3: a) Confocal microscopy imaging of **$C_{8'}TC_{12}$** and **$C_{14}TC_{12}$** (300 µM) stained with lipophilic styryl dye, and histogram of the size distribution of (b) **$C_{8'}TC_{12}$** and (c) **$C_{14}TC_{12}$**, and histogram of the size distribution in presence of siRNA of (d) **$C_{8'}TC_{12}$** and (e) **$C_{14}TC_{12}$** from confocal microscopy images.

6. Esterase-induced degradation

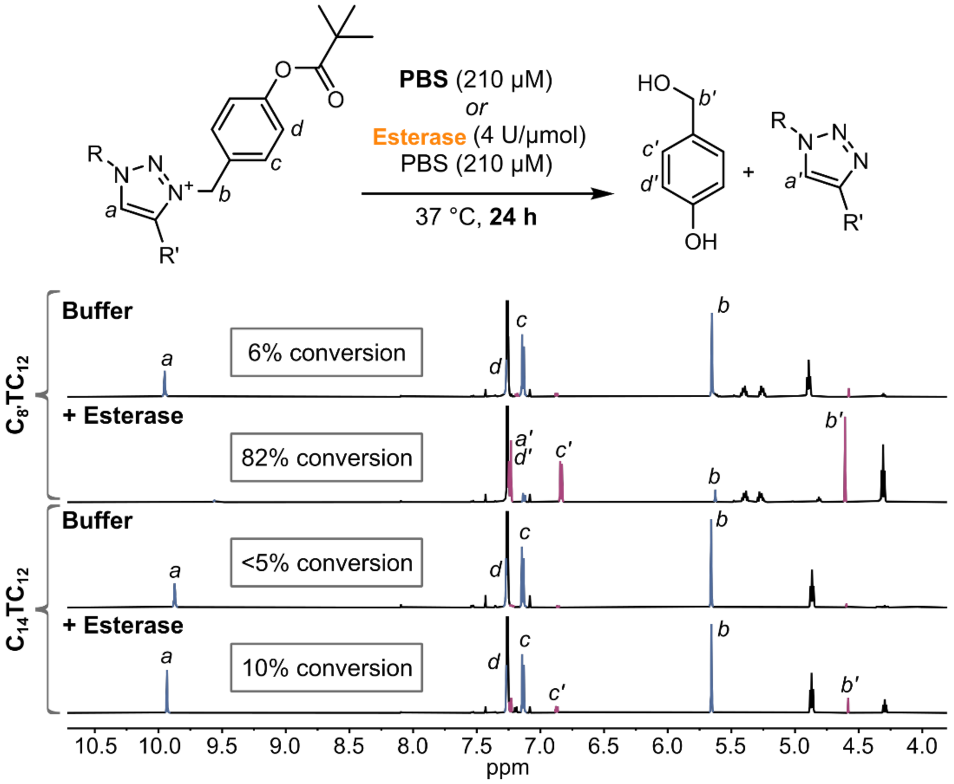

Figure S4: ^{1}H NMR stacked plot of products after stirring compounds **C8'TC12** (top) and **C14TC12** (bottom) at 37 °C for 24 hours in the absence (upper) or in the presence of esterase (lower).

7. Ethidium Bromide Displacement Assay

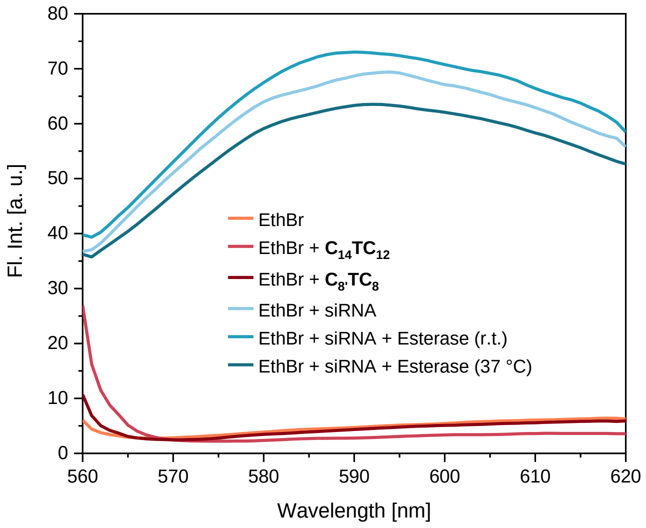

Figure S5: Fluorescence spectra of ethidium bromide (5 µM) admixed with triazolium compounds $\mathbf{C_{8'}TC_{12}}$ and $\mathbf{C_{14}TC_{12}}$ (210 µM), siRNA (0.5 µM) and esterase (4 U/µmol) in PBS buffer. Excitation wavelength was set to 546 nm.

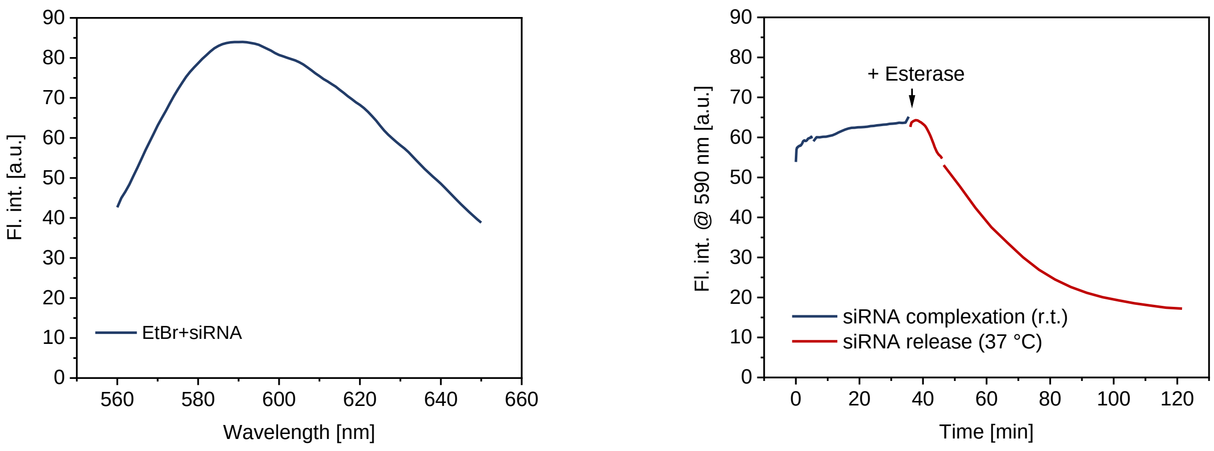

Figure S6: Left: Fluorescence spectrum of a solution of ethidium bromide (5 µM) and siRNA (0.5 µM) in PBS. Right: Kinetic fluorescence monitoring of Method 2: a solution of ethidium bromide (5 µM) and $\mathbf{C_{8'}TC_{12}}$ (210 µM) was equilibrated for 10 minutes. siRNA (0.5 µM) was added and the recording was started. After 40 minutes, the fluorimeter was heated to 37 °C and esterase (4 U/µmol) was added. First recording after approximately one minute shows decrease in fluorescence of around 20 a. u. compared to spectrum with EthBr/siRNA complex (same measurement conditions).

8. Dynamic Light Scattering and ζ-Potential

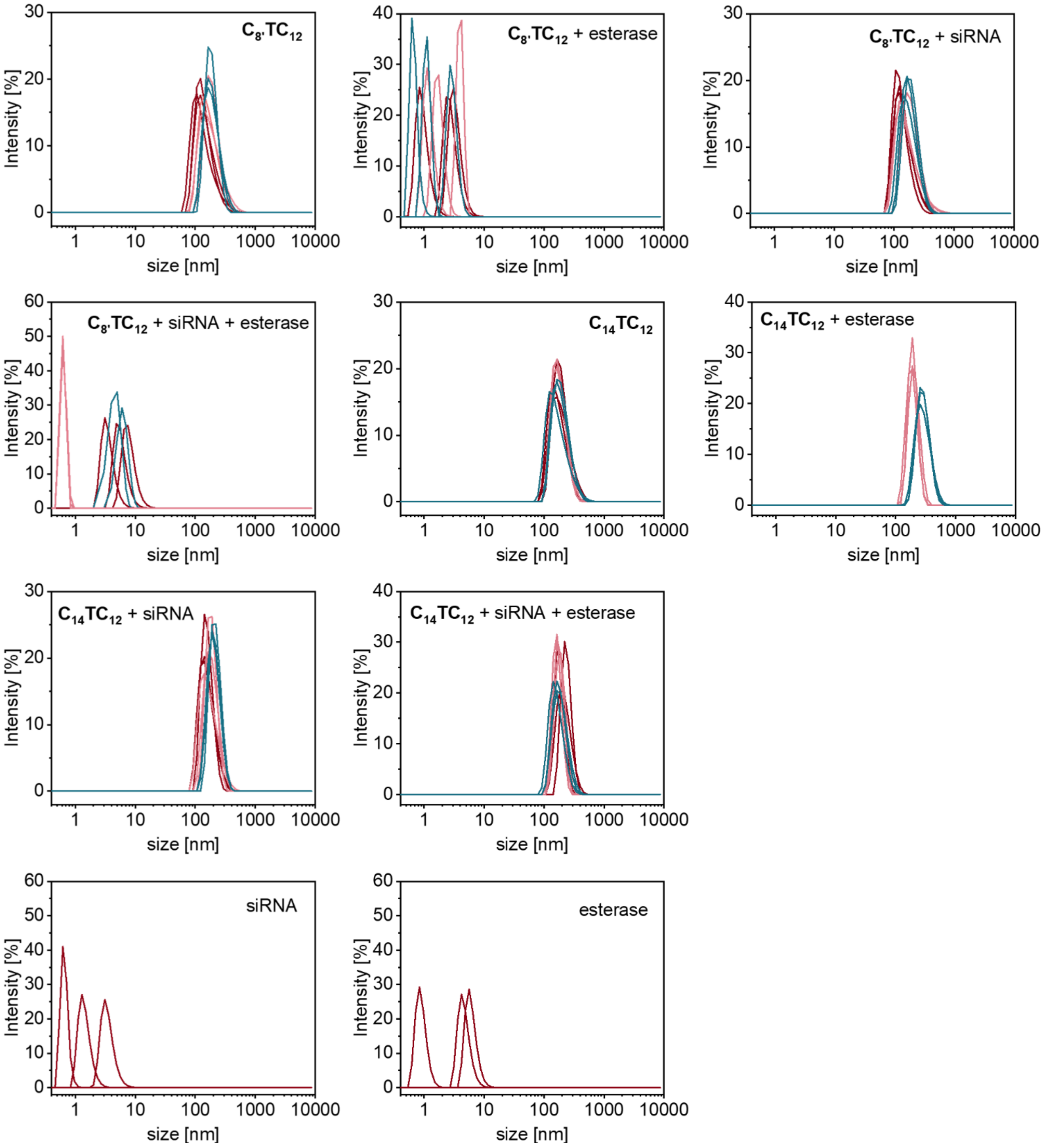

Figure S7: The hydrodynamic diameters of aggregates of triazolium compounds **$C_{8'}TC_{12}$** and **$C_{14}TC_{12}$** (30 µM) with siRNA (70 nM) and/or esterase (4 U/µmol), and pristine siRNA and esterase in PBS buffer. One color depicts three measurement with 10 runs (7 seconds) each. Those measurements were performed in triplicate with different batches.

Table S2: Mean Count Rate of DLS measurements of **$C_{8'}TC_{12}$** and **$C_{14}TC_{12}$** after the addition of siRNA and/or esterase in PBS buffer.

Compounds	Mean Count Rate ± SD	Attenuator	Measurement position
**$C_{8'}TC_{12}$**	1190 ± 111	11	3
**+ Esterase**	25 ± 1	11	3
**+ siRNA**	1190 ± 8	11	3
**+ siRNA + Esterase**	23 ± 1	11	3
**$C_{14}TC_{12}$**	639 ± 21	11	3
**+ Esterase**	232 ± 8	11	4.65
**+ siRNA**	410 ± 15	11	4.65
**+ siRNA + Esterase**	193 ± 7	11	4.65

Table S3: Particle size, polydispersity index (PDI) and ζ-potential characterization by dynamic light scattering and ζ-potential measurements of pristine siRNA (70 nM in PBS), esterase (0.12 U/mL) and PBS buffer. Measured in triplicate. n. d. = not determined.

Compounds	Size ± SD (nm)	PDI ± SD	ζ-potential ± SD (mV)
**siRNA**	1.8 ± 0.1	0.66 ± 0.04	0.01 ± 1.6
**Esterase**	2.3 ± 1.4	0.77 ± 0.21	-0.39 ± 2.24
**PBS buffer**	n. d.	n. d.	-1.32 ± 2.56

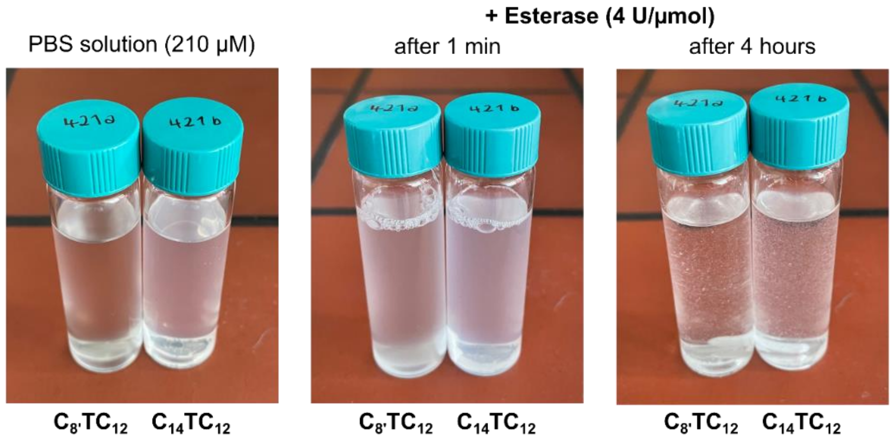

Figure S8: Clear to slightly turbid PBS solutions of compounds **$C_{8'}TC_{12}$** and **$C_{14}TC_{12}$** (left), turbid solution directly after addition of esterase (middle) and after 4 hours at 37 °C with precipitating triazole compounds (right).

9. NMR Spectra

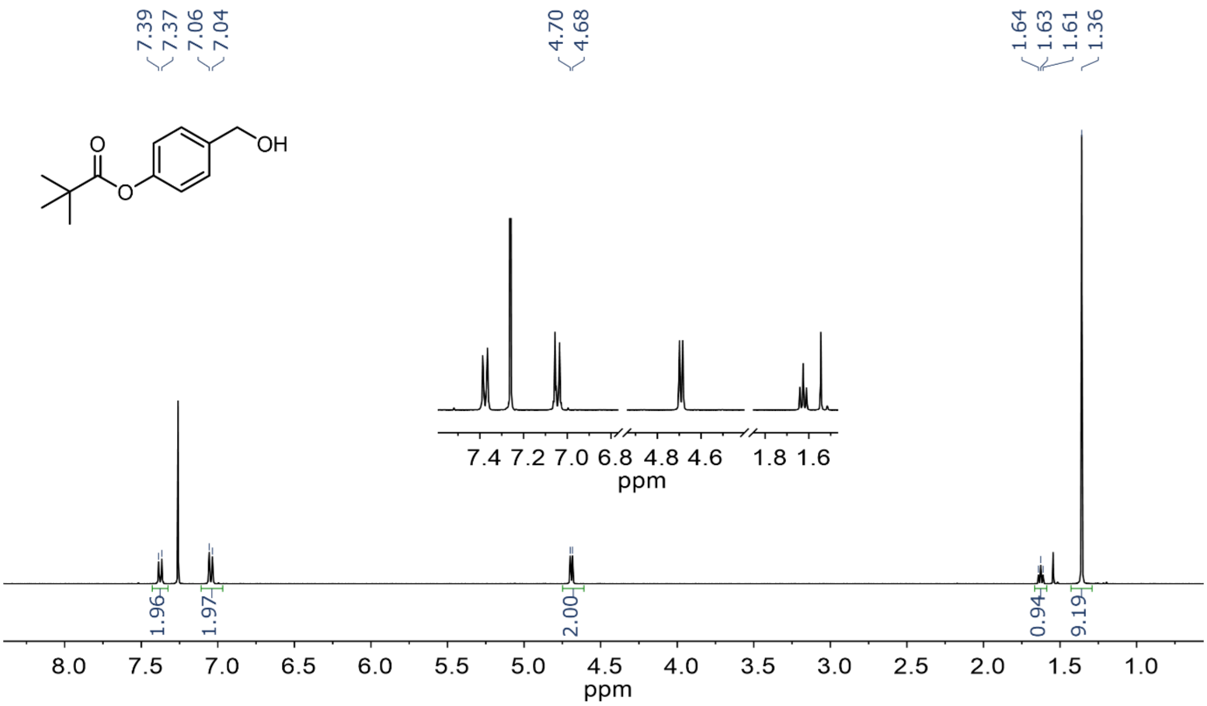

Figure S9: ^{1}H NMR ($CDCl_3$, 400 MHz, 293 K) spectrum of compound (**S2**).

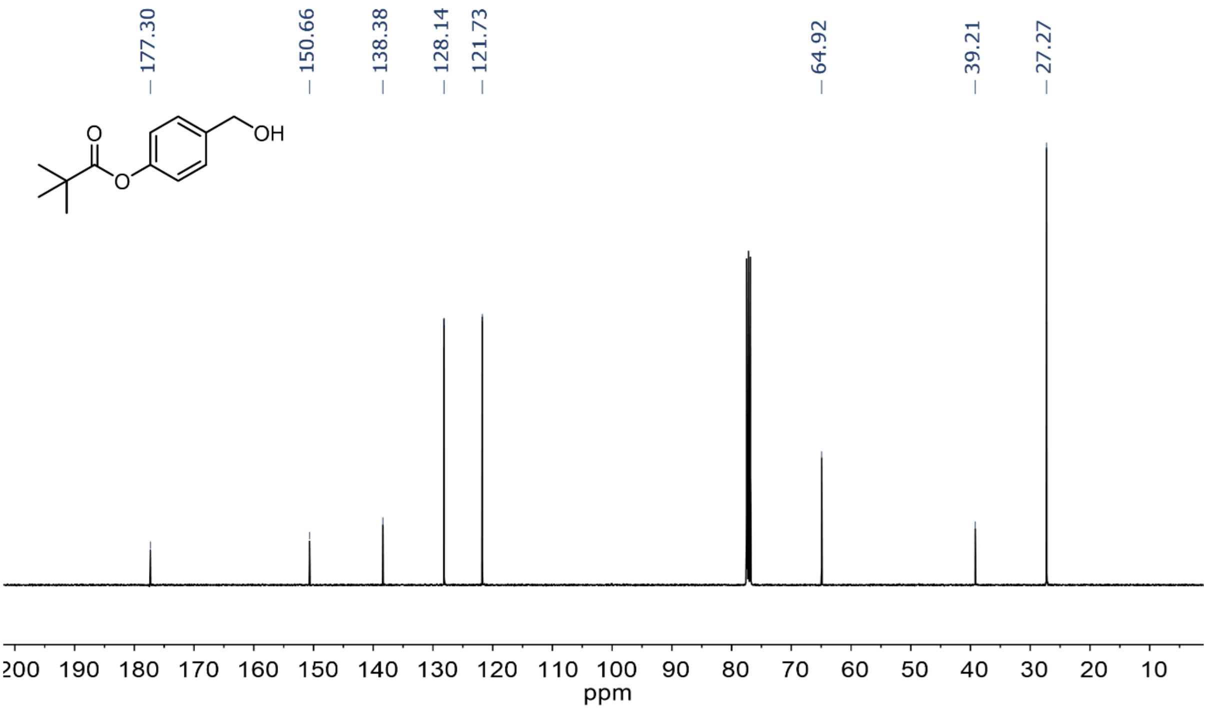

Figure S10: ^{13}C NMR ($CDCl_3$, 101 MHz, 293 K) spectrum of compound (**S2**).

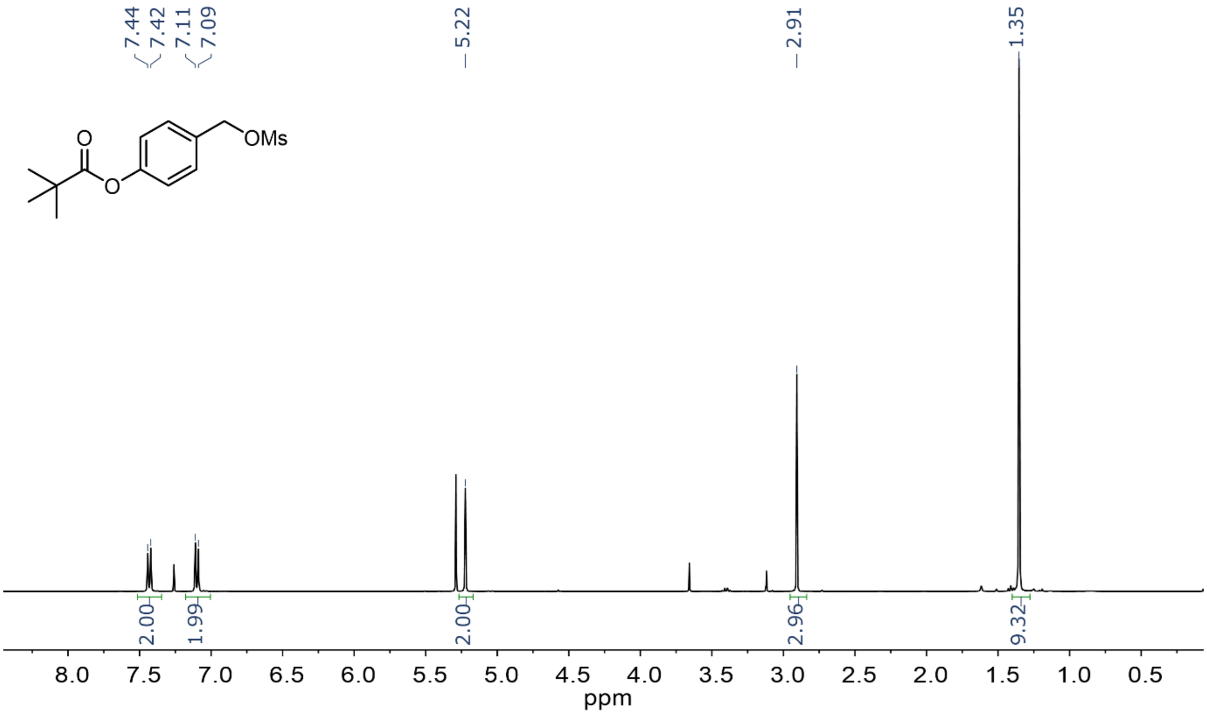

Figure S11: ^{1}H NMR ($CDCl_3$, 400 MHz, 293 K) spectrum of compound (**S3**).

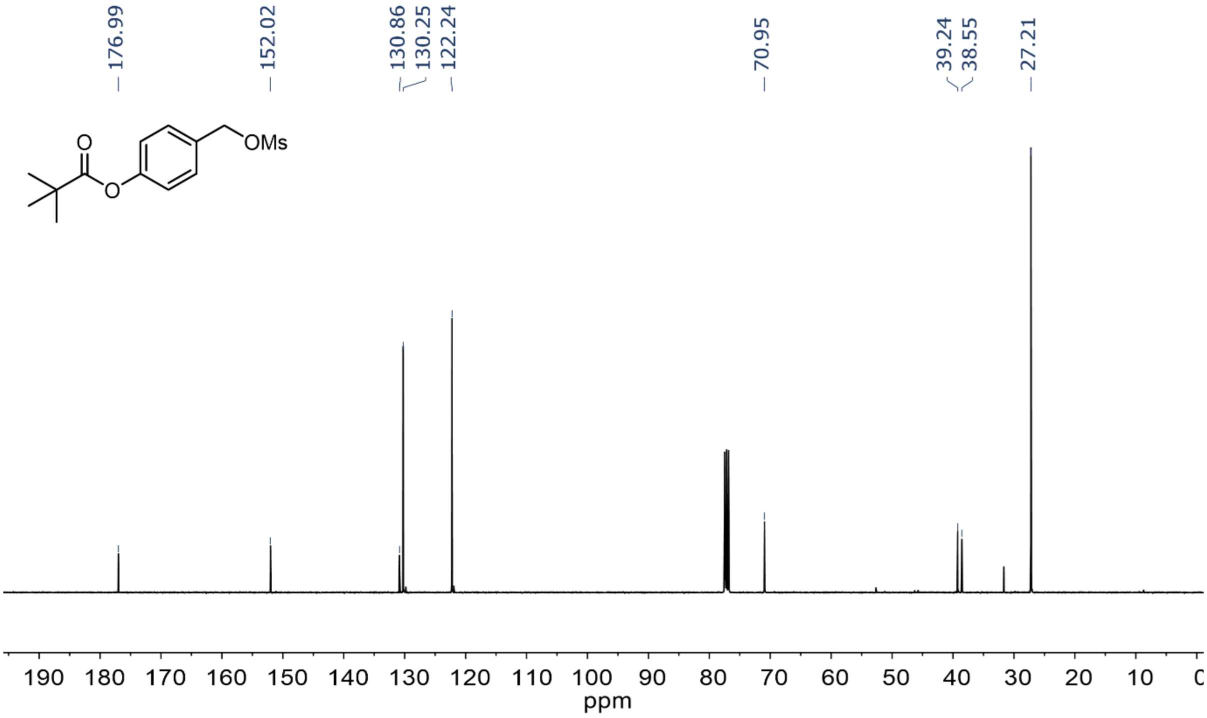

Figure S12: ^{13}C NMR ($CDCl_3$, 101 MHz, 293 K) spectrum of compound (**S3**).

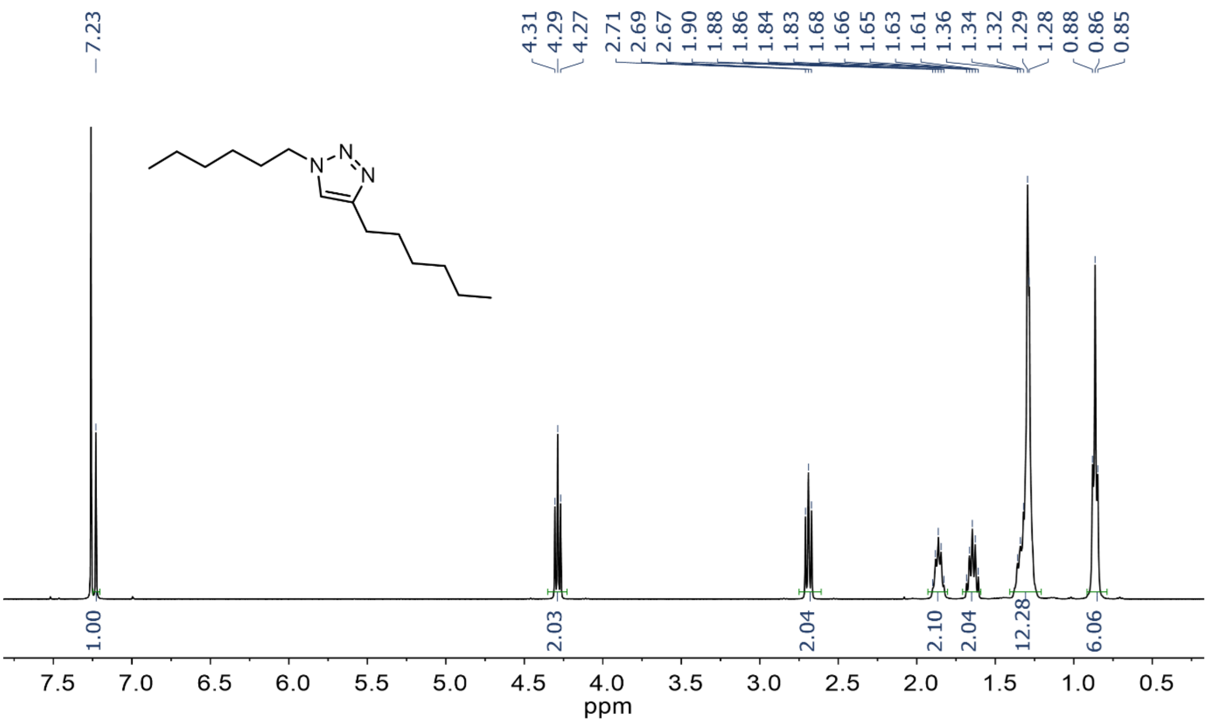

Figure 13: ^{1}H NMR ($CDCl_3$, 400 MHz, 293 K) spectrum of compound (**S7a**).

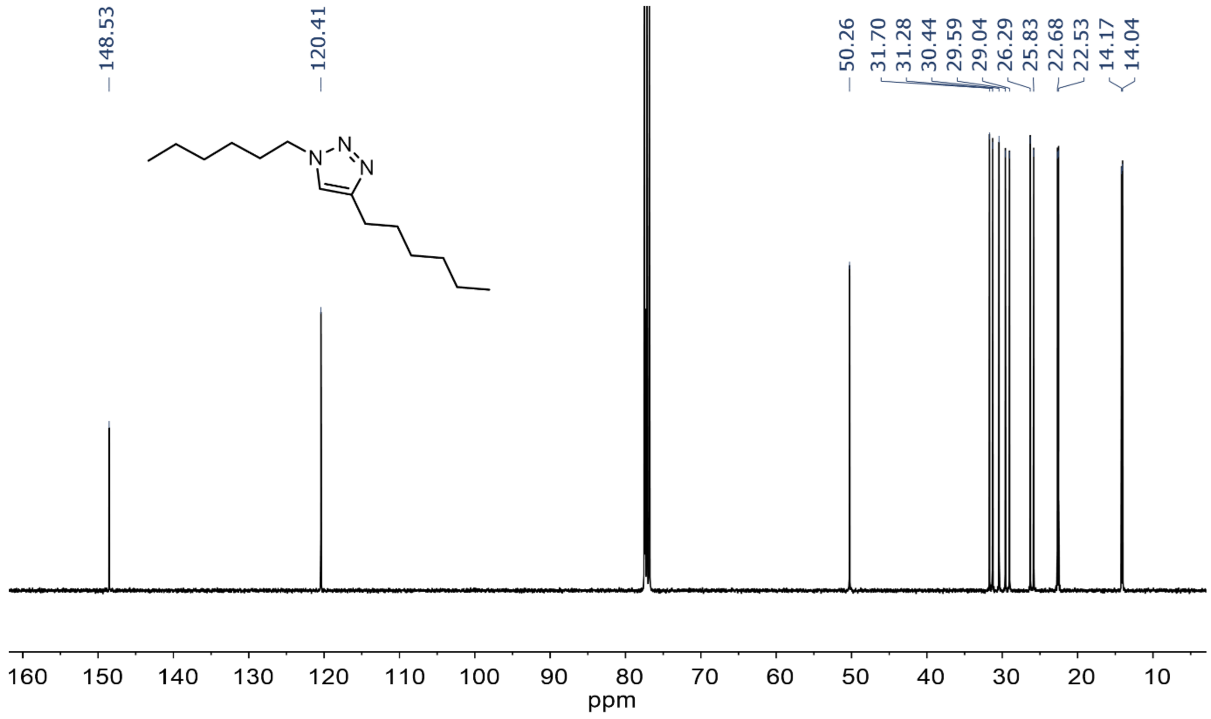

Figure S14: ^{13}C NMR ($CDCl_3$, 101 MHz, 293 K) spectrum of compound (**S7a**).

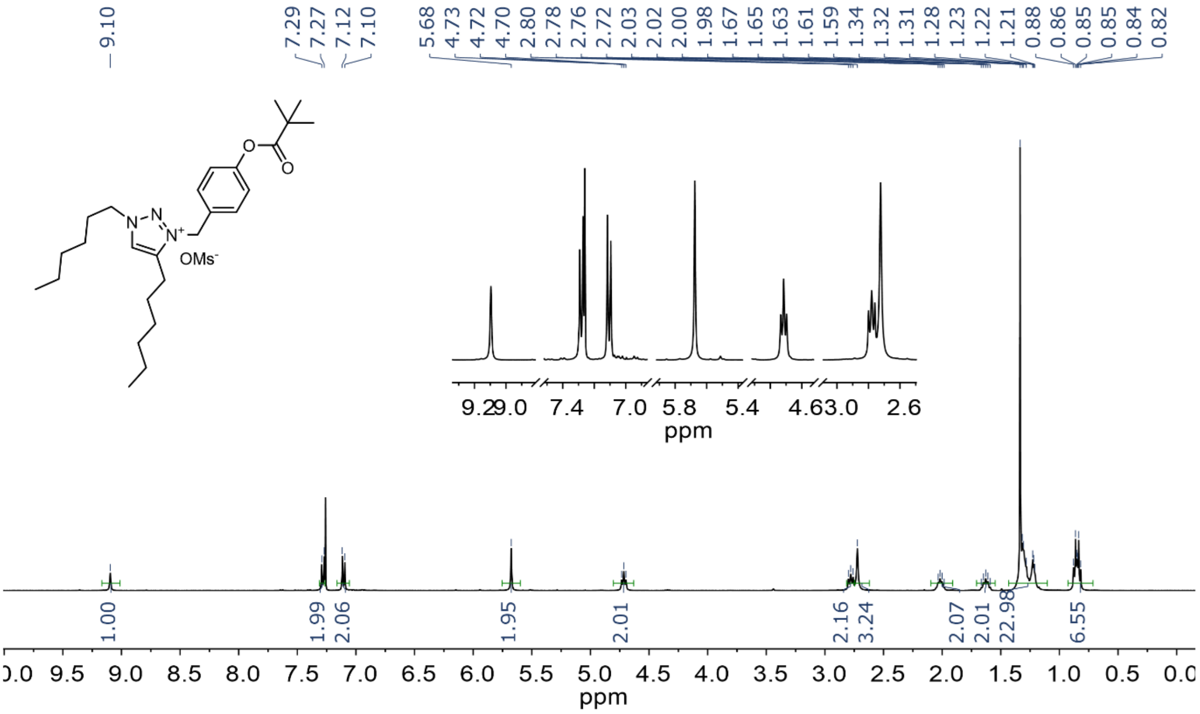

Figure S15: ^{1}H NMR ($CDCl_3$, 400 MHz, 293 K) spectrum of compound (**C_6TC_6**).

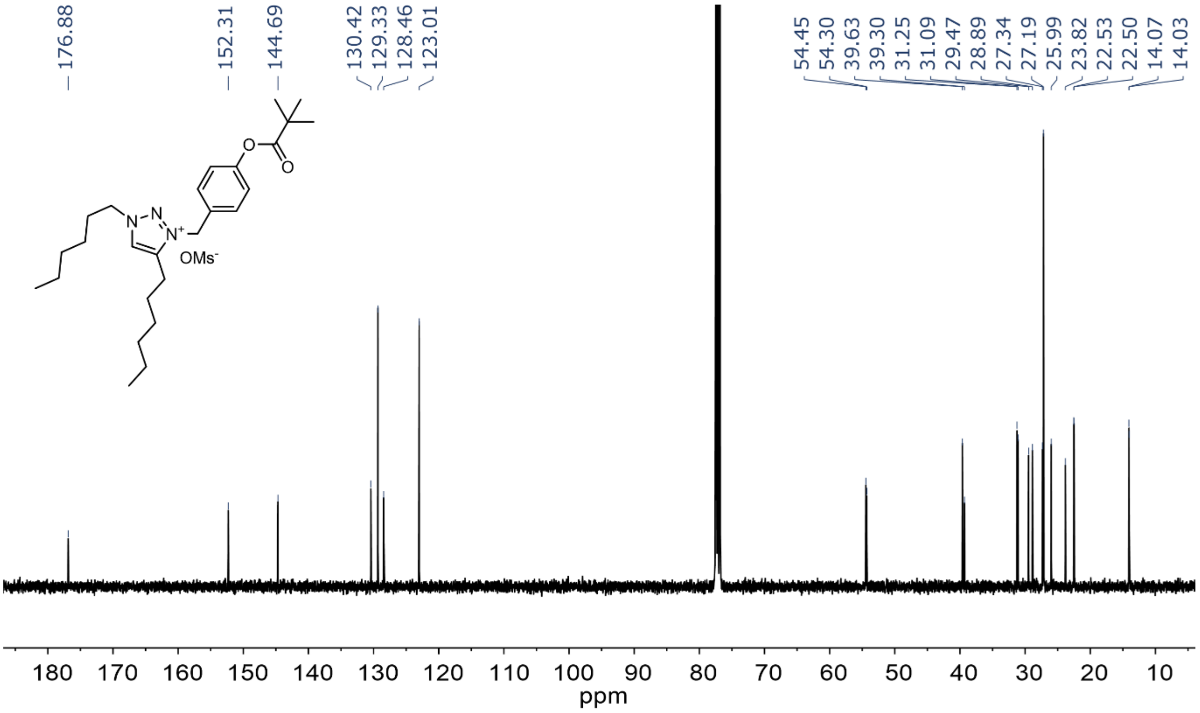

Figure S16: ^{13}C NMR ($CDCl_3$, 101 MHz, 293 K) spectrum of compound (**C_6TC_6**).

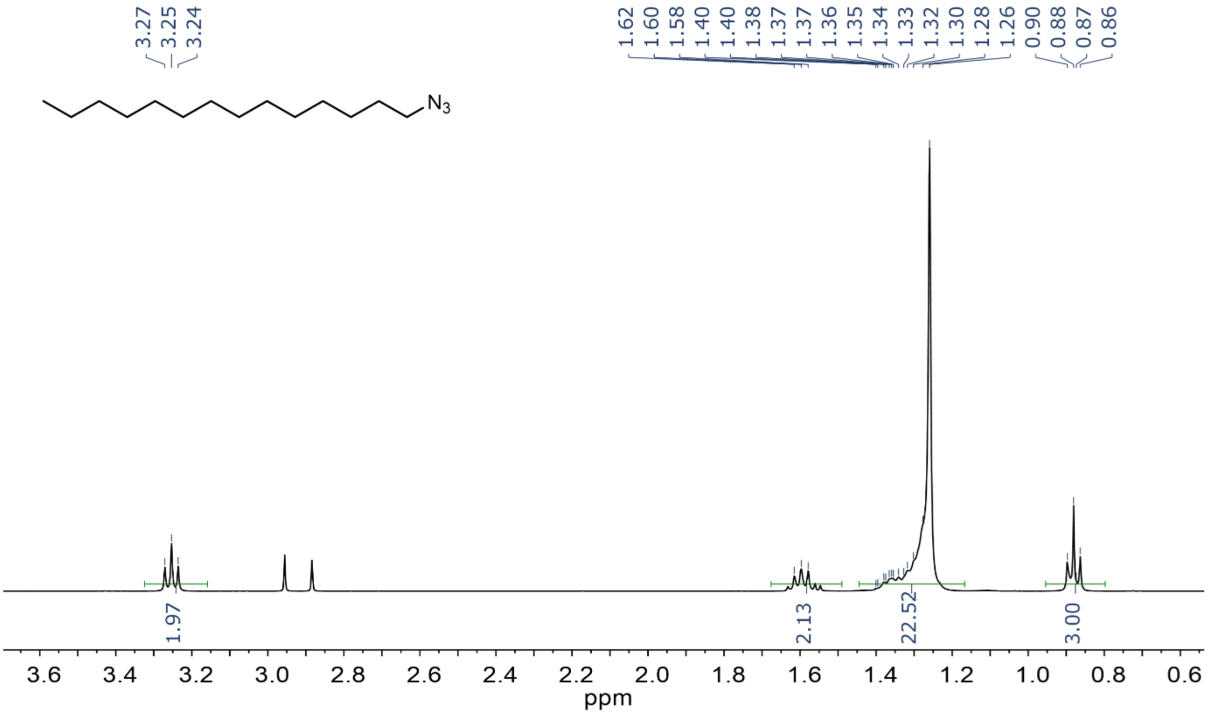

Figure S17: ^{1}H NMR ($CDCl_3$, 400 MHz, 293 K) spectrum of compound (**S6b**).

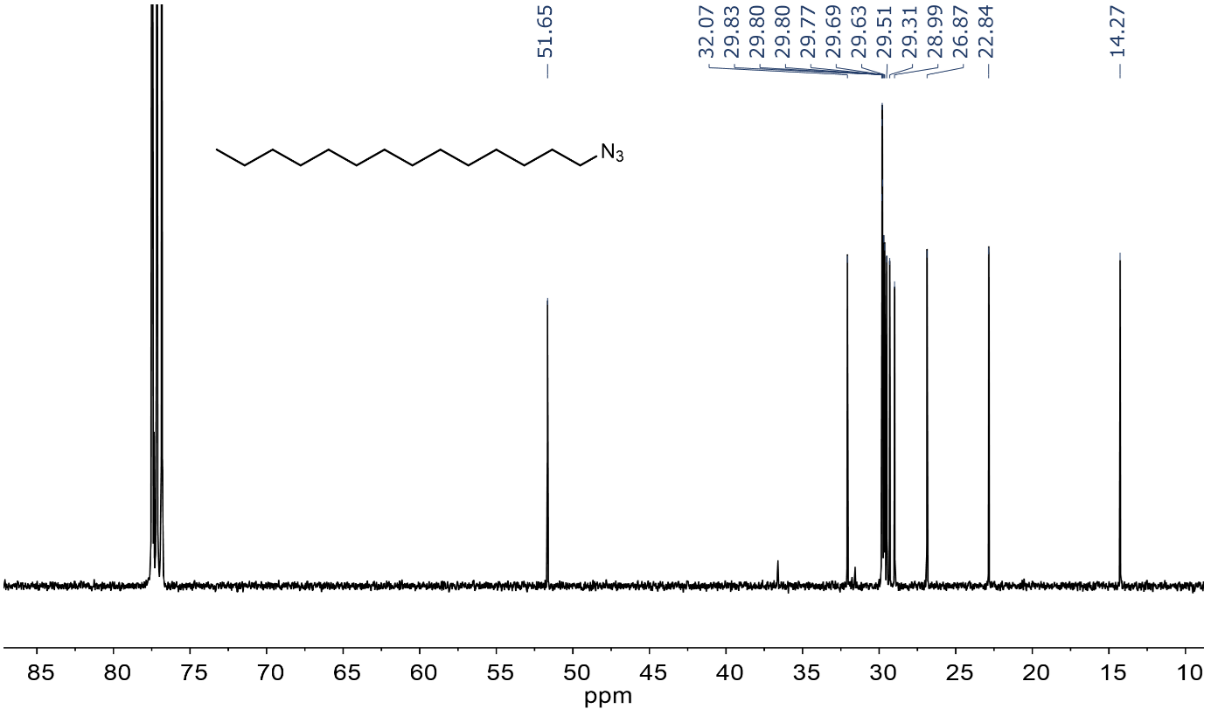

Figure 18: ^{13}C NMR ($CDCl_3$, 101 MHz, 293 K) spectrum of compound (**S6b**).

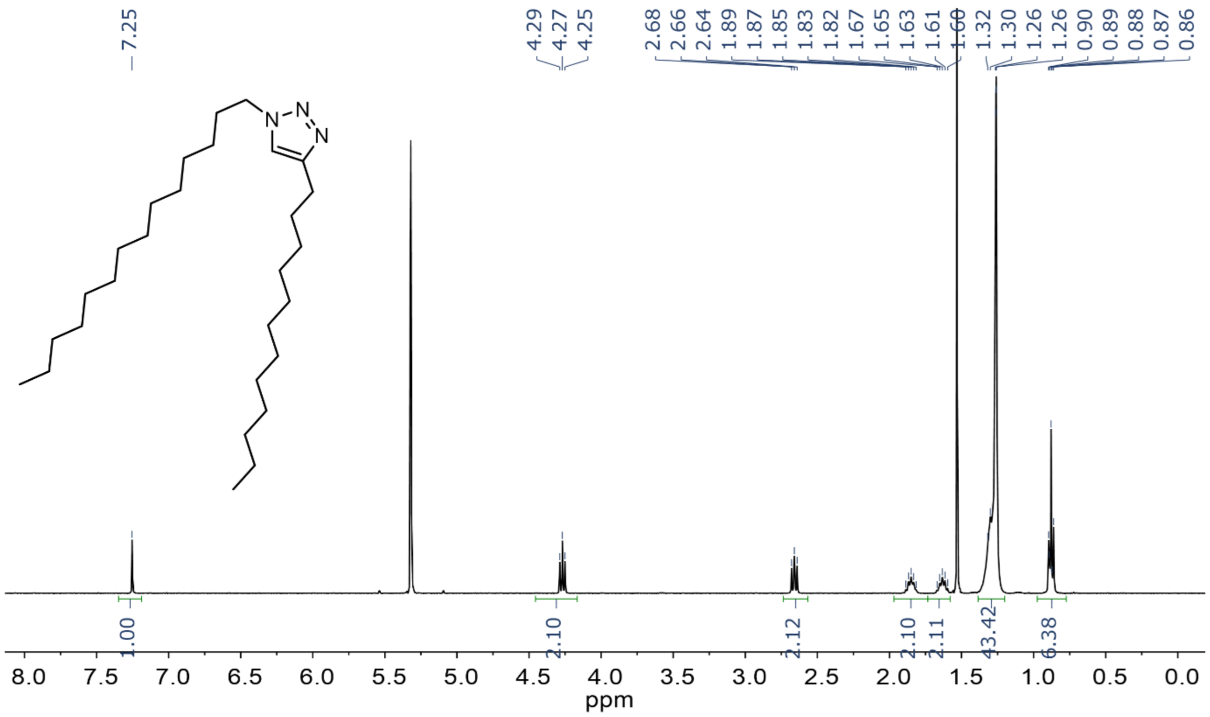

Figure S19: ^{1}H NMR (CD_2Cl_2, 400 MHz, 293 K) spectrum of compound (**S7b**).

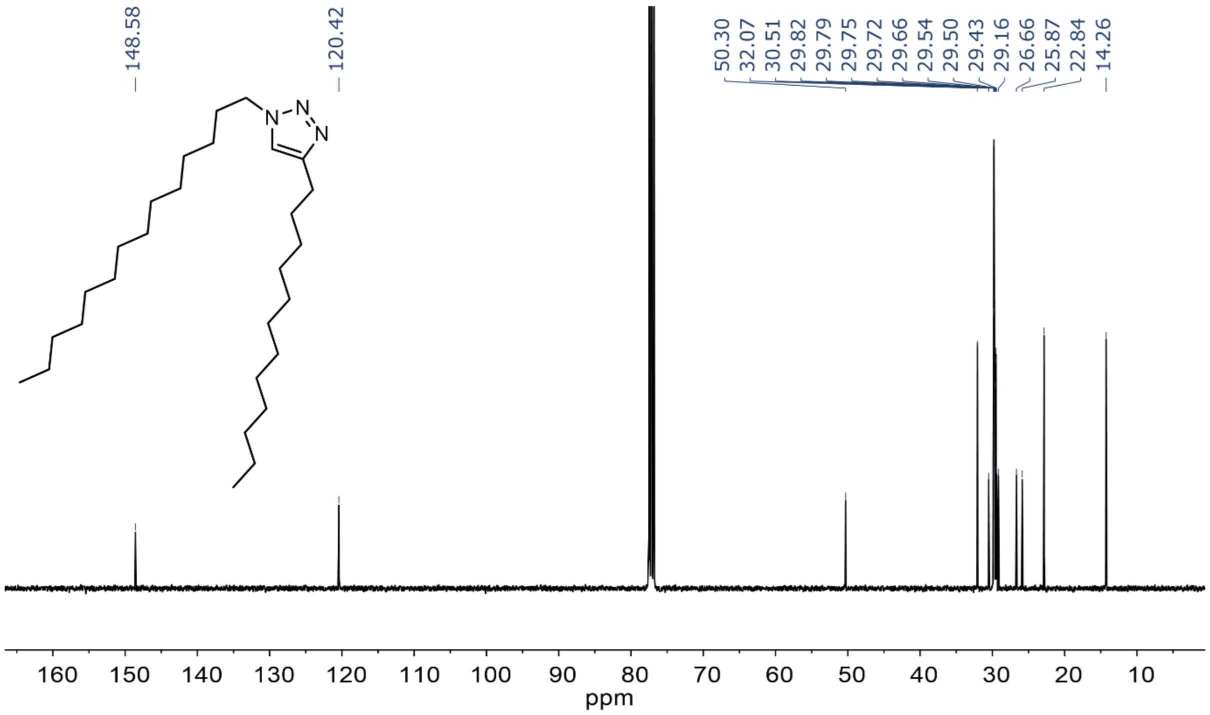

Figure S20: ^{13}C NMR ($CDCl_3$, 101 MHz, 293 K) spectrum of compound (**S7b**).

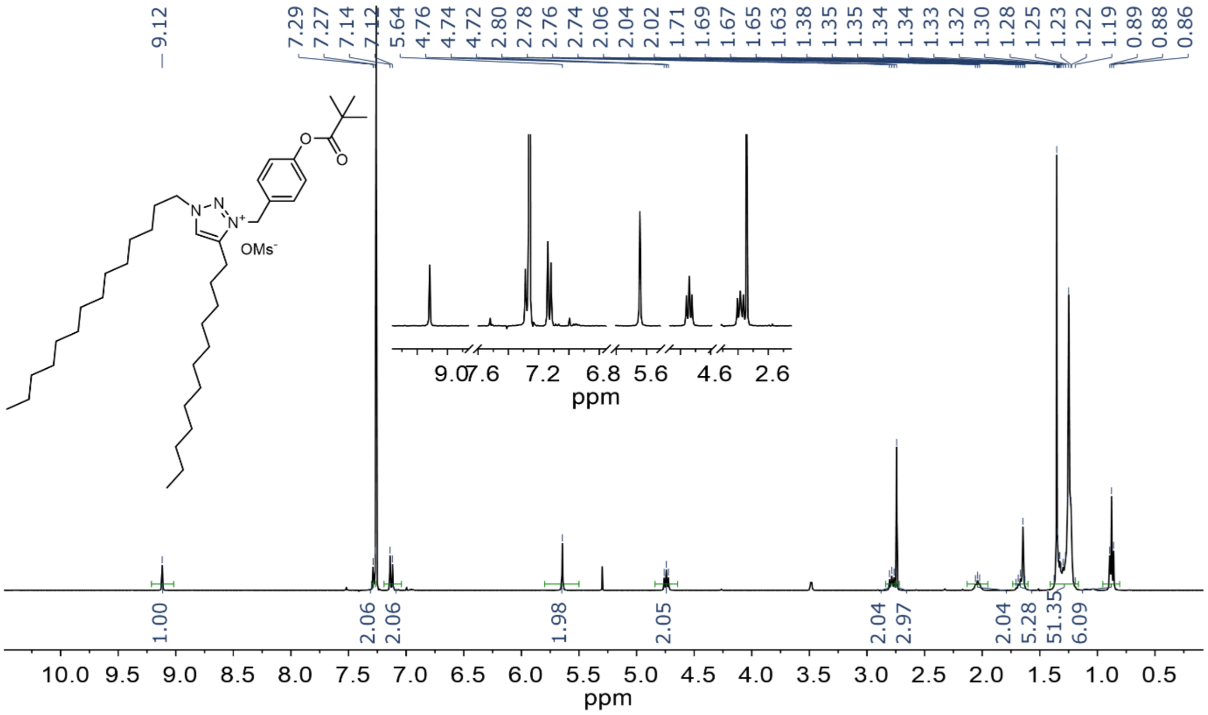

Figure S21: ^{1}H NMR ($CDCl_3$, 400 MHz, 293 K) spectrum of compound (**$C_{14}TC_{12}$**).

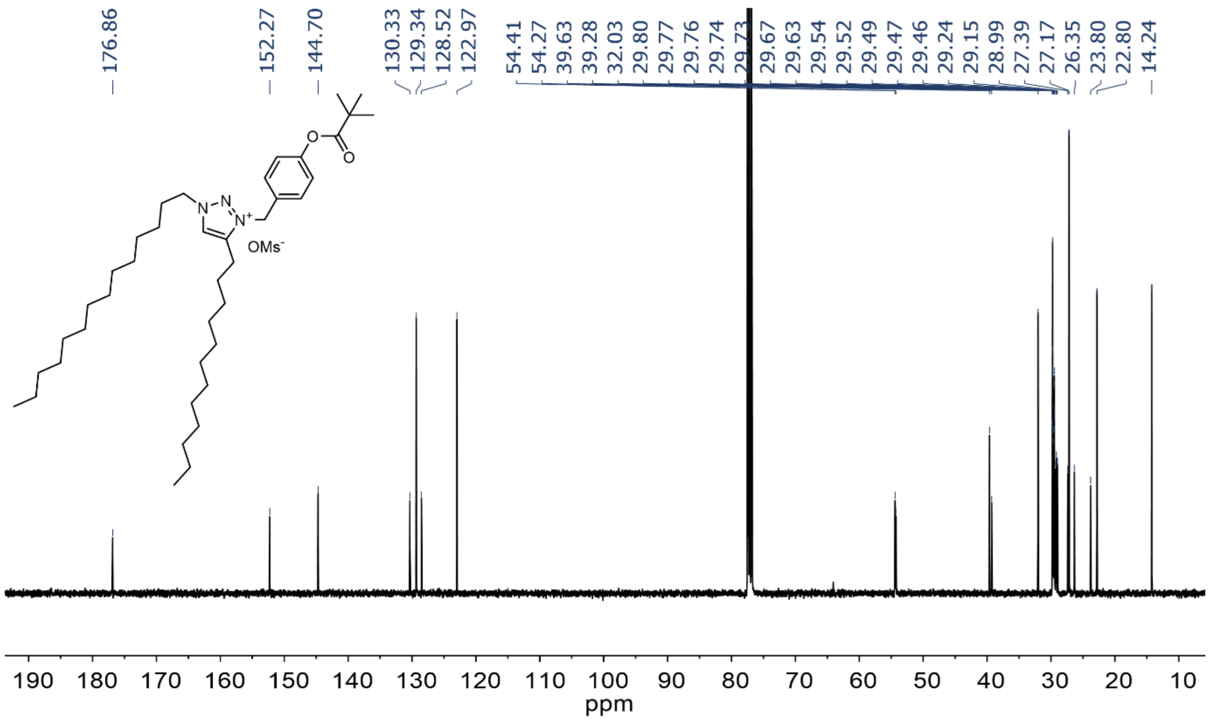

Figure S22: ^{13}C NMR ($CDCl_3$, 101 MHz, 293 K) spectrum of compound (**$C_{14}TC_{12}$**).

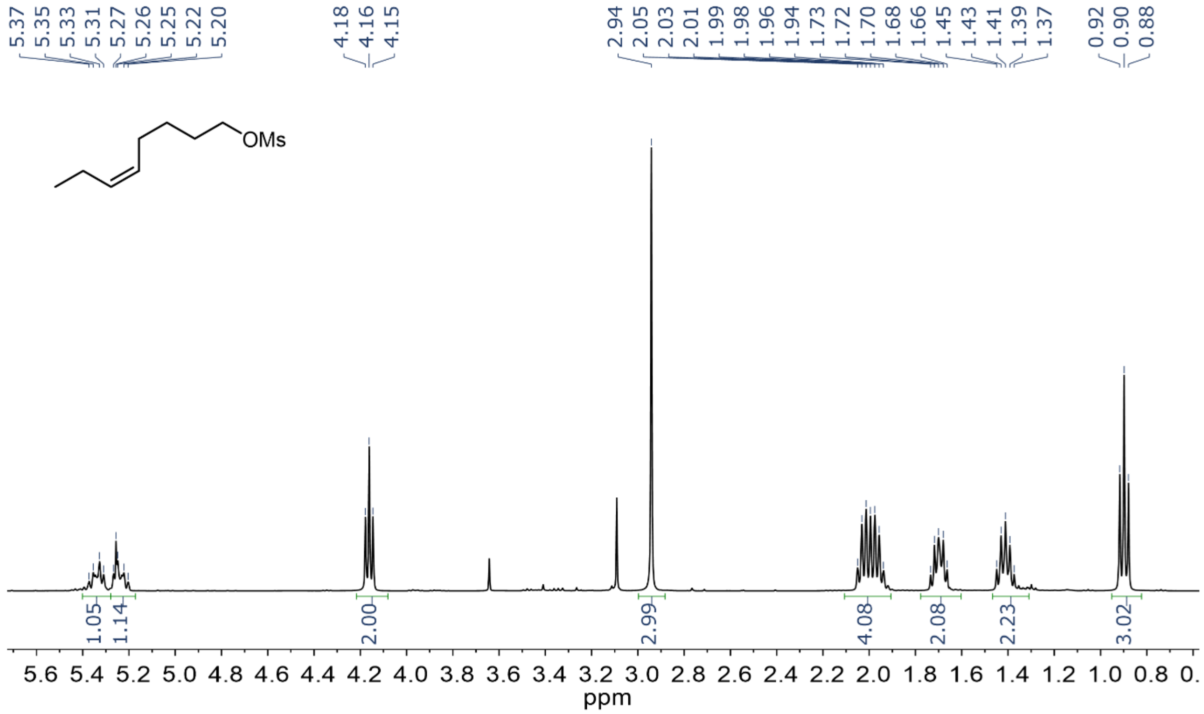

Figure S23: ^{1}H NMR ($CDCl_3$, 400 MHz, 293 K) spectrum of compound (**S5c**).

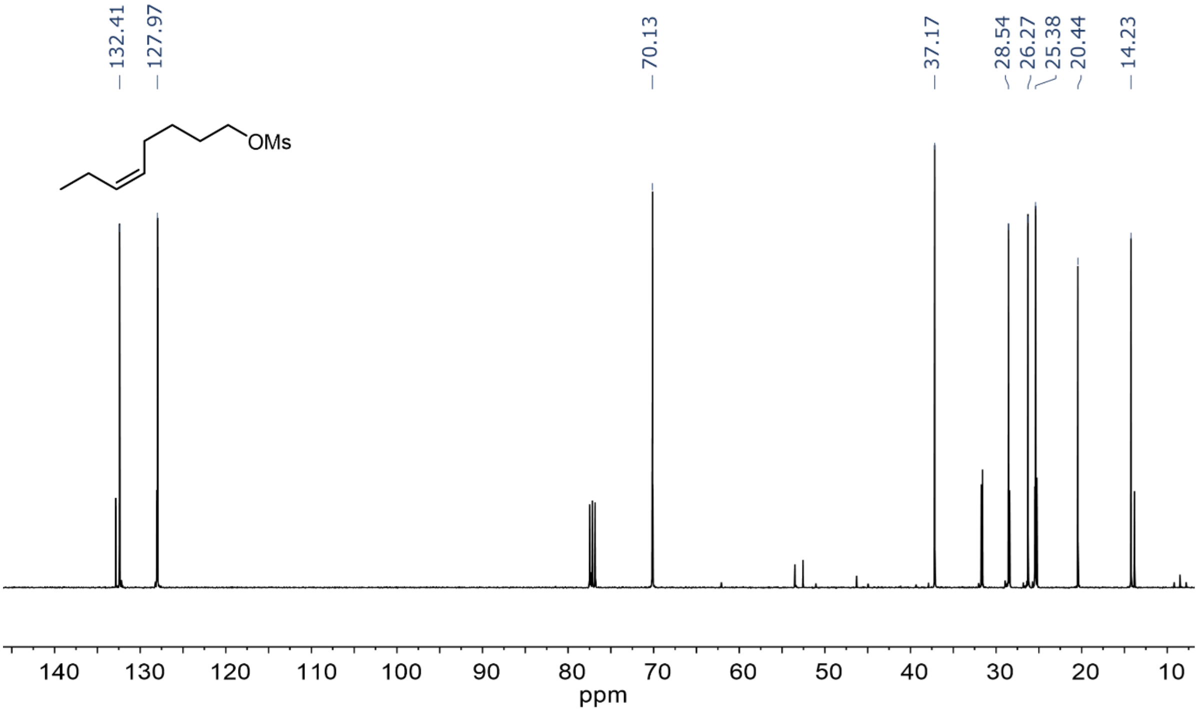

Figure S24: ^{13}C NMR ($CDCl_3$, 101 MHz, 293 K) spectrum of compound (**S5c**).

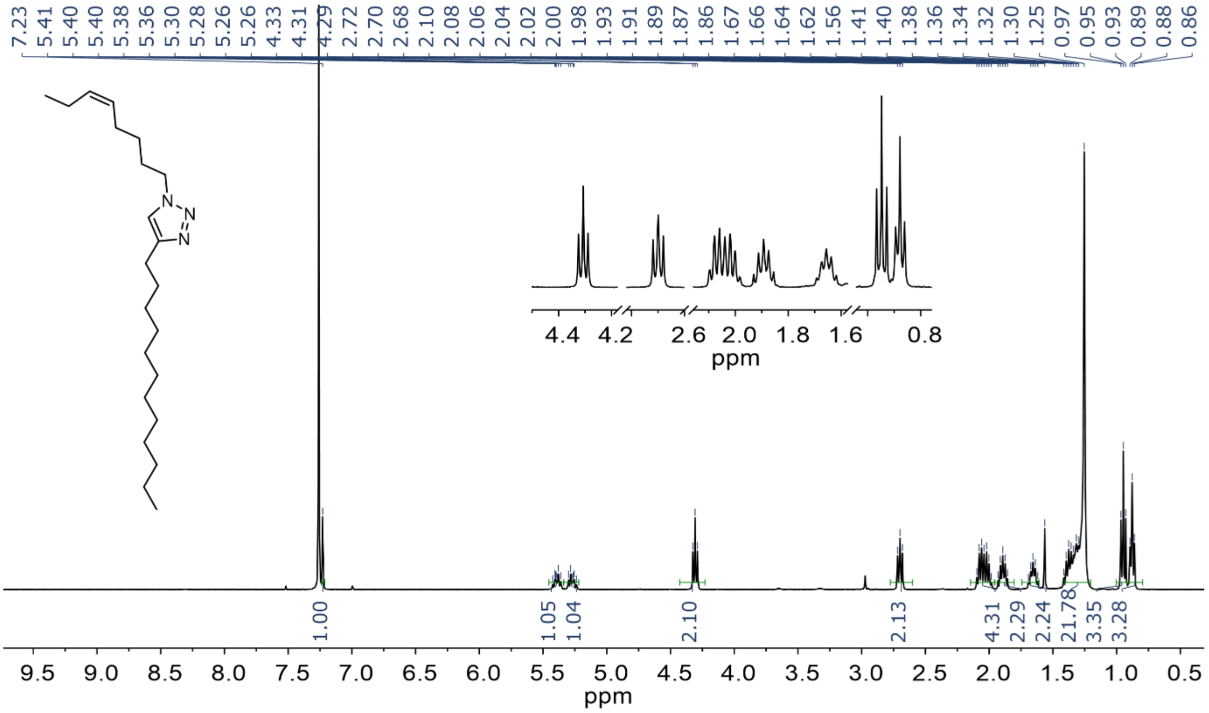

Figure S25: ^{1}H NMR ($CDCl_3$, 400 MHz, 293 K) spectrum of compound (**S7c**).

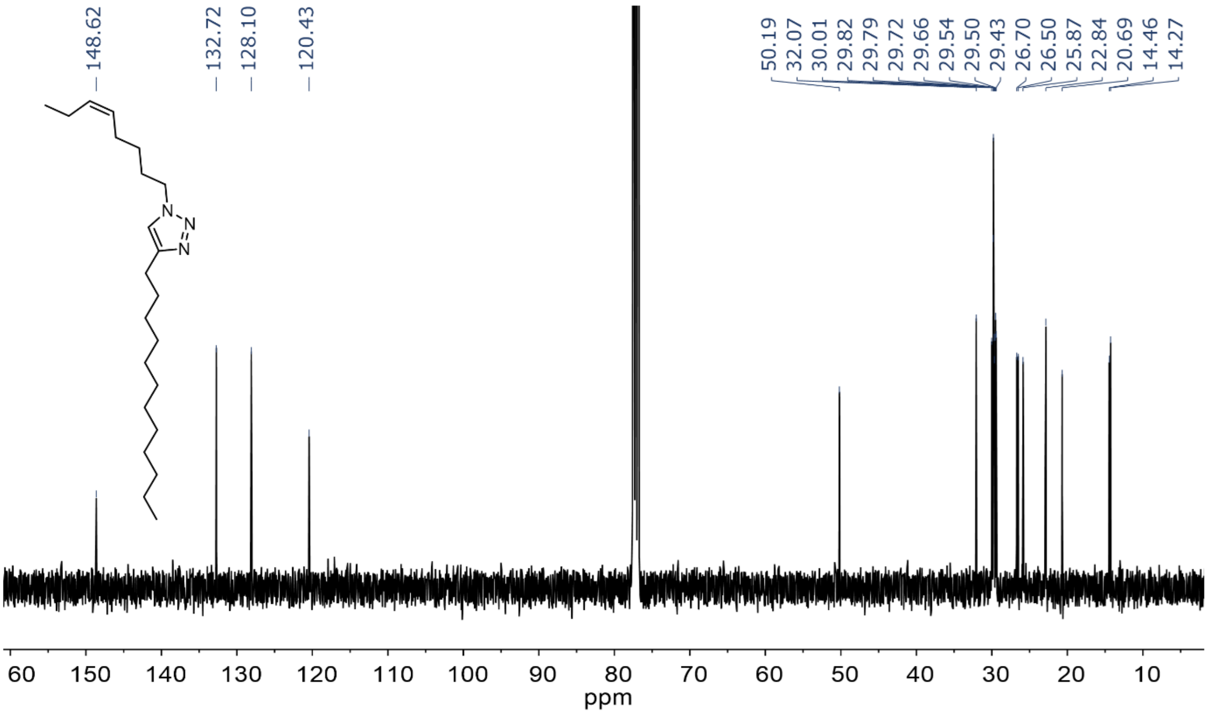

Figure S26: ^{13}C NMR ($CDCl_3$, 101 MHz, 293 K) spectrum of compound (**S7c**).

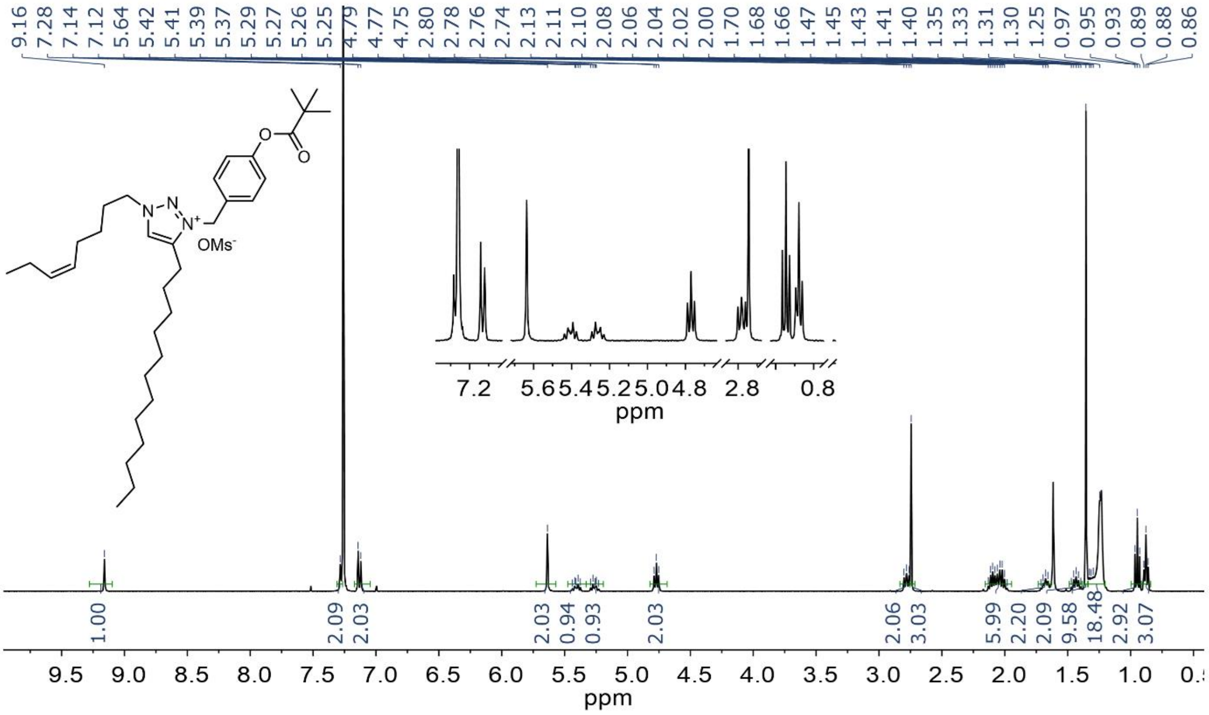

Figure S27: ^{1}H NMR ($CDCl_3$, 400 MHz, 293 K) spectrum of compound (**$C_{8'}TC_{12}$**).

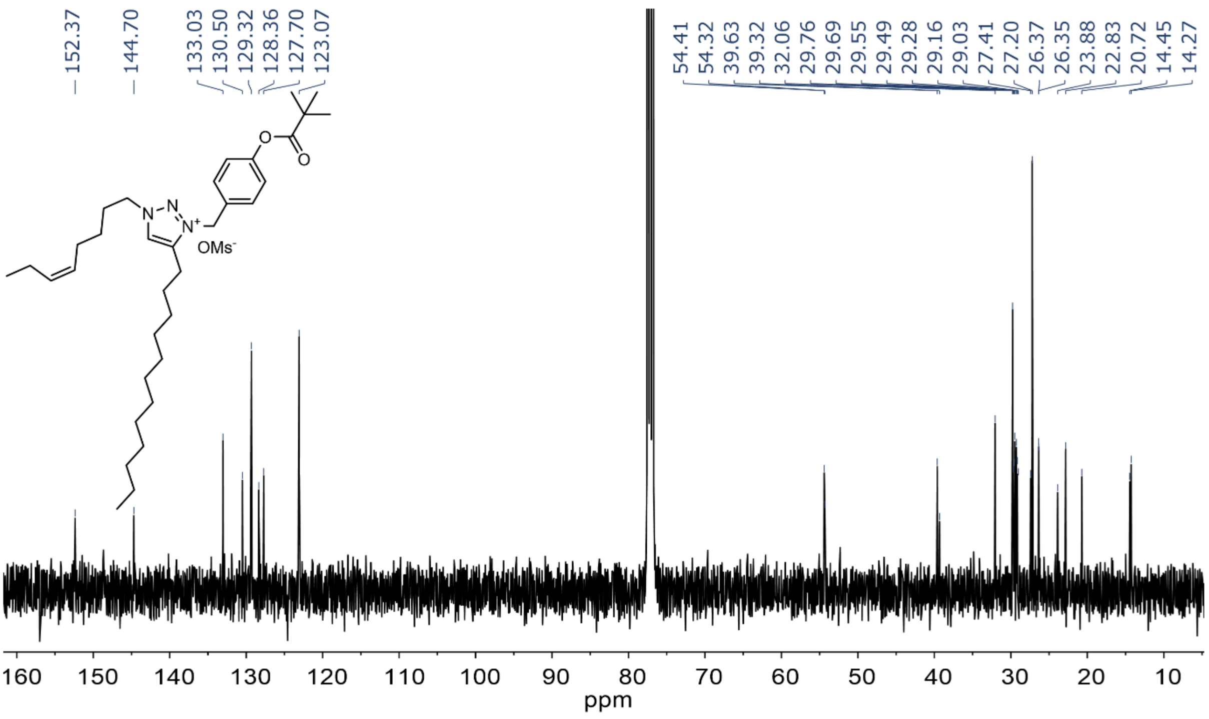

Figure S28: ^{13}C NMR ($CDCl_3$, 101 MHz, 293 K) spectrum of compound (**$C_{8'}TC_{12}$**).
